# TRIM28‐Derived Peptide Exerts Anti‐Tumor Roles by Stabilizing Tumor Suppressive BRD7 Protein in Multiple Cancers

**DOI:** 10.1002/advs.76974

**Published:** 2026-08-05

**Authors:** Qingqing Wei, Changning Xue, Mengna Li, Jianxia Wei, Lemei Zheng, Shipeng Chen, Yijie Wang, Yumei Duan, Yanwei Luo, Wei Xiong, Ming Zhou

**Affiliations:** ^1^ NHC Key Laboratory of Carcinogenesis Hunan Key Laboratory of Oncotarget Gene Hunan Cancer Hospital and the Affiliated Cancer Hospital of Xiangya School of Medicine Central South University Changsha China; ^2^ Cancer Research Institute and School of Basic Medical Sciences Central South University Changsha China; ^3^ The Key Laboratory of Carcinogenesis and Cancer Invasion of the Chinese Ministry of Education Central South University Changsha China; ^4^ Xiangya School of Medicine Central South University Changsha China; ^5^ Department of Blood Transfusion The Third Xiangya Hospital Central South University Changsha Hunan China

**Keywords:** anti‐tumor, BRD7, multiple cancers, peptide, targeted therapy, TRIM28

## Abstract

BRD7 serves as a critical tumor suppressor, yet it is frequently inactivated via ubiquitination‐mediated proteasomal degradation, facilitating tumor progression and metastasis. Although stabilizing or activating BRD7 represents a promising therapeutic strategy, pharmacological agents to achieve this remain limited. Here, we aimed to develop a peptide‐based approach to enhance BRD7 protein stability. We first mapped the TRIM28‐BRD7 interaction to the 299–349 aa region within the Coiled‐coil domain of TRIM28. Based on this interface, we designed two α‐helical peptides, TAB12 and TAB14, that mimic the key structural features. Mechanistically, TAB12 exhibits strong binding affinity toward BRD7 protein and competitively disrupts the TRIM28‐BRD7 interaction, thereby blocking TRIM28‐mediated BRD7 ubiquitination and stabilizing endogenous BRD7. In vitro functional assays demonstrated that TAB12 markedly inhibits tumor cell proliferation, migration, and invasion, induces cell apoptosis, and presents low cytotoxicity toward normal cells. Subsequent rescue experiments confirmed that BRD7 knockdown substantially abrogates the anti‐tumor effects of TAB12, verifying that TAB12's function depends on stabilizing BRD7. Furthermore, in vivo animal models validated that TAB12 significantly suppresses tumor growth with a favorable safety profile. Therefore, this study identifies a novel peptide agent that exerts broad anti‐tumor effects by stabilizing BRD7, offering a feasible strategy for restoring tumor suppressor function.

## Graphical abstract

An α‐helical peptide, TAB12, designed to mimic the TRIM28 binding interface, competitively disrupts the TRIM28‐BRD7 interaction, thereby blocking ubiquitin‐mediated degradation of the tumor suppressor BRD7. This stabilization unleashes potent anti‐tumor effects across multiple tumor types with a favorable safety profile, offering a feasible strategy for restoring tumor suppressor function in cancer therapy.

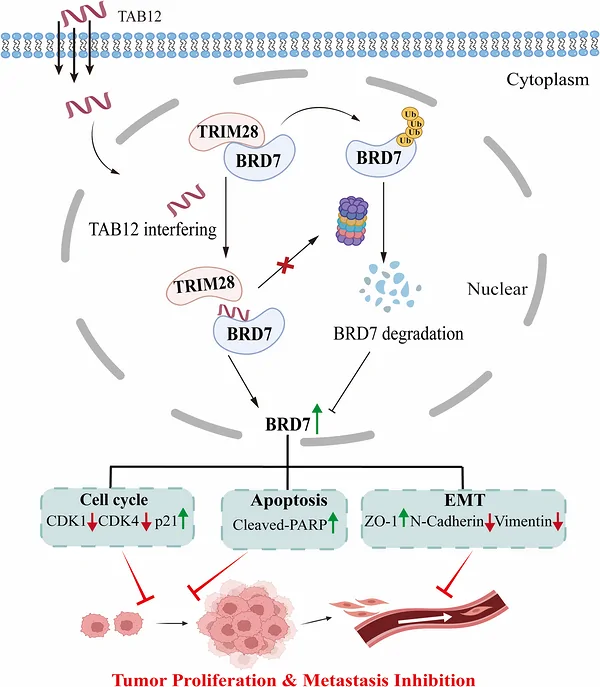

## 1 Introduction

Bromodomain‐containing protein 7 (BRD7) was first identified and cloned in our laboratory as a gene significantly downregulated in nasopharyngeal carcinoma [1]. The BRD7 protein mainly localizes in the nucleus and contains two defining structural features: an evolutionarily conserved bromodomain (BRD) and a functional nuclear localization signal (NLS) [2]. Subsequent studies have demonstrated that BRD7 expression is frequently downregulated in diverse malignancies, including breast cancer [3], liver cancer [4], gastric cancer [5], ovarian cancer [6], and prostate cancer [7]. Functioning as a tumor suppressor, BRD7 exerts its anti‐oncogenic effects through multiple mechanisms. Specifically, it arrests the cell cycle at the G1/S transition, induces apoptosis, suppresses invasive and metastatic behaviors, and sensitizes tumors to radiotherapy and chemotherapy [8, 9, 10]. Furthermore, the expression level of BRD7 is negatively correlated with tumor malignant progression and poor patient prognosis. Taken together, these data establish that loss of BRD7 function is a key driver of tumor malignancy and poor clinical outcomes, highlighting BRD7 as an attractive candidate for cancer therapy with significant clinical translational value.

The tripartite motif‐containing (TRIM) family comprises a group of highly conserved RING‐type E3 ubiquitin ligases, including over 80 members identified in the human genome [11]. TRIM28 (also known as KAP1 or TIF1β) is a central member of this family, playing a crucial role in transcriptional regulation and chromatin remodeling, primarily through its interaction with KRAB zinc‐finger proteins [12, 13]. Structurally, TRIM28 contains the canonical RBCC (RING, B‐Box, and Coiled‐coil) domains at its N‐terminus, along with a unique C‐terminal PHD domain and bromodomain. Accumulating evidence indicates that TRIM28 is frequently overexpressed in multiple cancer types, including hepatocellular carcinoma [14], non‐small cell lung cancer [15], breast cancer [16], gastric cancer [17], and prostate cancer [18]. It drives tumor progression by directly regulating core oncogenic processes, including cell cycle, apoptosis, metastasis, stemness maintenance, and immune evasion. In our prior study, we employed immunoprecipitation‐mass spectrometry (IP‐MS) screening to identify TRIM28 as a potential E3 ubiquitin ligase targeting BRD7. We further demonstrated that TRIM28 interacts with BRD7 and promotes breast cancer malignancy by mediating BRD7 ubiquitination and subsequent proteasomal degradation [19]. These findings suggest that aberrant TRIM28/BRD7 signaling is a critical molecular mechanism that drives breast cancer progression.

Protein‐protein interactions (PPIs) constitute a core mechanism underlying virtually all biological processes, and their abnormal activation is a hallmark of various malignant tumors. Consequently, specifically targeting aberrantly activated PPIs within oncogenic signaling pathways has emerged as a promising therapeutic strategy against tumors [20, 21]. Despite rapid advancements in small‐molecule drugs and biologics, they struggle to efficiently bind PPI interfaces that are characterized by large and flat surface areas. Peptide drugs, composed of 5–50 amino acid residues, exhibit unique advantages for targeting PPI interfaces [22]. Peptides derived from natural PPI interfaces or their analogues can competitively occupy binding sites by mimicking key structural features, thereby disrupting protein‐protein interactions and further influencing the stability or function of target proteins [23, 24]. Compared to other drugs, peptide therapeutics exhibit higher target specificity and a generally favorable immunogenicity profile, which helps minimize off‐target effects on normal tissues and improves treatment safety [25, 26]. Over the past decade, an increasing number of anti‐tumor peptides and peptidomimetics targeting PPIs have been developed and applied. Notable examples include MDM2‐p53 targeting peptides that restore p53 activity [27], PD‐1/PD‐L1 blocking peptides that alleviate immune suppression [28], peptide inhibitors targeting the METTL3/METTL14 complex, and peptides disrupting the Nrf2/Keap1 interaction [29, 30], all of which have shown significant progress. Therefore, peptide compounds targeting protein interactions have great potential for application in the field of tumor therapy.

In this study, we designed a novel peptide inhibitor, TAB12, based on the binding interface between BRD7 and its E3 ubiquitin ligase TRIM28. TAB12 competitively binds BRD7, blocking its recognition and ubiquitination by TRIM28 and thereby preventing BRD7 degradation. By stabilizing the BRD7 protein, TAB12 inhibited the proliferation, invasion, and migration of multiple cancer cell lines, induced apoptosis, and demonstrated potent anti‐tumor activity and a favorable safety profile in vivo. This study introduces a peptide inhibitor that reactivates the tumor suppressor BRD7 by disrupting its interaction with TRIM28. Rather than inhibiting an oncogenic driver, this strategy works by directly stabilizing a tumor suppressor protein.

## 2 Results

### 2.1 Design of Anti‐Tumor Peptides Targeting and Stabilizing the BRD7 Protein

Having confirmed the interaction between TRIM28 and BRD7 in breast cancer, we next determined whether this regulatory relationship is conserved in other cancer types. We first examined the protein expression profiles of TRIM28 and BRD7 across a panel of cell lines, including breast cancer (MDA‐MB‐231, MCF7), nasopharyngeal carcinoma (CNE2), hepatocellular carcinoma (Hep3B), and normal epithelial cells (MCF10A, NP69), and revealed that TRIM28 was upregulated and BRD7 downregulated in cancer cells compared with normal cells (Figure S1A). Subsequently, Co‐IP assays were performed, and specific binding between TRIM28 and BRD7 was detected in both CNE2 and Hep3B cells, consistent with our previous observations in breast cancer cells (Figure S1B). This finding strongly suggests that such an interaction may be prevalent across different types of tumor cells. To identify the key interacting domain between TRIM28 and BRD7 protein, we utilized a series of Flag‐tagged TRIM28 domain‐deletion mutants, including ΔRING, ΔB‐BOX, ΔCoiled‐coil, ΔPHD, and ΔBROMO (Figure 1A). These mutants, along with the HA‐BRD7 plasmid, were co‐transfected into the breast cancer cell line MCF7. Subsequently, Co‐IP and Western blot analyses revealed that only deletion of the Coiled‐coil (CC) domain of TRIM28 blocked the TRIM28‐BRD7 interaction (Figure 1B), indicating that the Coiled‐coil domain of TRIM28 is the structural basis for its interaction with BRD7. To further validate this conclusion, we constructed a TRIM28 truncation variant containing only the Coiled‐coil domain (TRIM28‐CC). Both forward and reverse Co‐IP assays confirmed that the Coiled‐coil domain of TRIM28 directly binds to the BRD7 protein (Figure 1C). Furthermore, overexpression of the TRIM28‐CC plasmid increased BRD7 protein levels (Figure 1D), likely by competing with endogenous TRIM28 for BRD7 binding and thereby preventing BRD7 degradation. These results suggest that the TRIM28 CC domain is a promising target for designing peptide inhibitors to stabilize the BRD7 protein.

**FIGURE 1:**
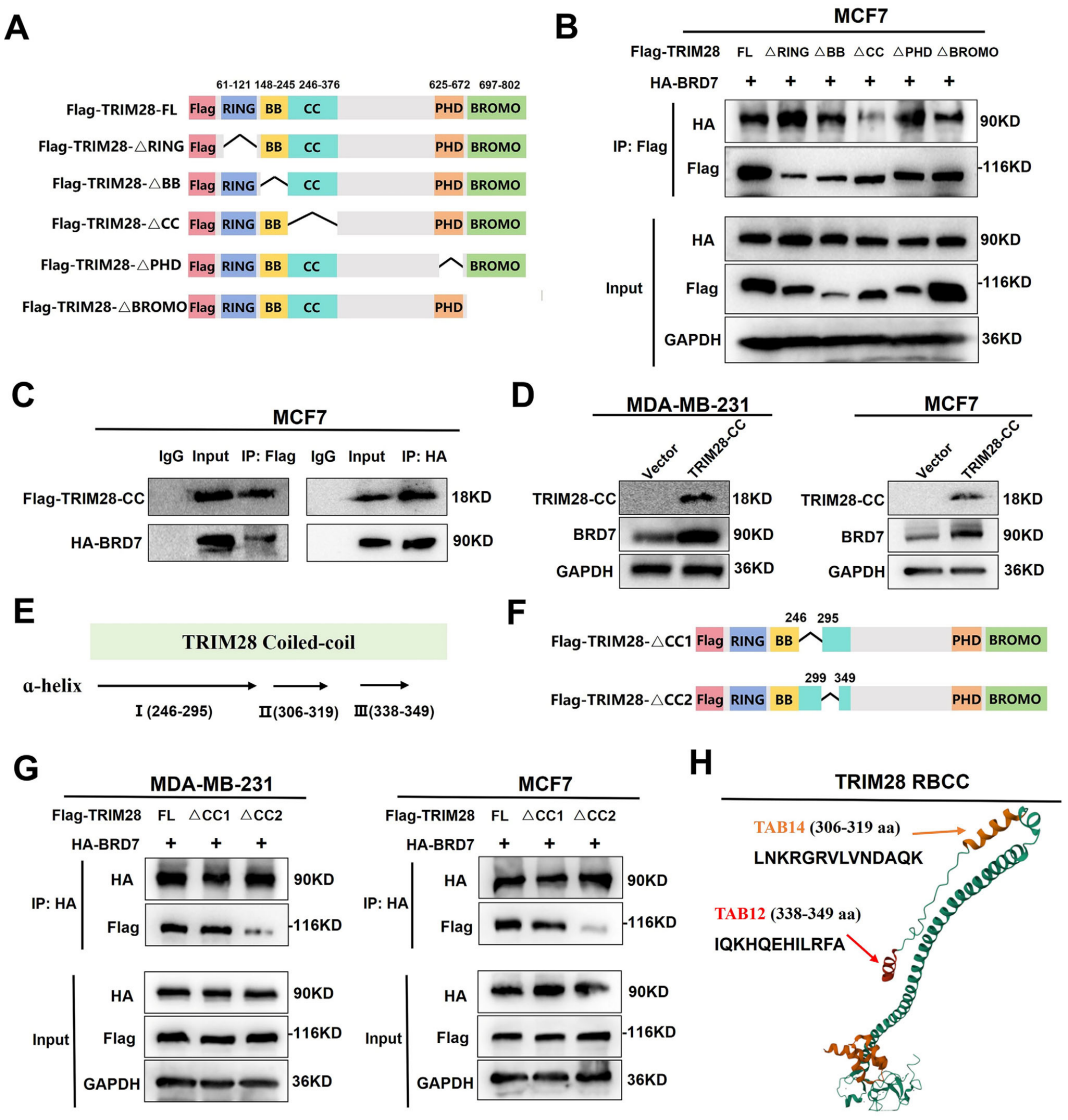
Design of anti‐tumor peptides targeting and stabilizing the BRD7 protein. (A) Schematic diagram of vectors encoding full‐length (FL) TRIM28 protein with a Flag tag and its various domain‐deletion mutants (ΔRING, ΔBB, ΔCC, ΔPHD, and ΔBROMO). (B) MCF7 cells were transfected with plasmids expressing either the FL or domain deletion mutants of TRIM28 along with HA‐BRD7. Subsequently, co‐immunoprecipitation was performed using an anti‐Flag antibody, followed by immunoblotting analysis. (C) After co‐transfecting MCF7 cells with Flag‐TRIM28‐CC (which only contains the coiled‐coil region) and HA‐BRD7 plasmids, forward and reverse co‐immunoprecipitation experiments were conducted using anti‐Flag and anti‐HA antibodies, respectively. Bound proteins were then detected by Western blotting using anti‐Flag and anti‐HA antibodies. (D) The expression levels of the BRD7 protein in the control cells and the cells overexpressing TRIM28‐CC were detected by Western blot. (E) Schematic diagram of the α‐helix within the Coiled‐coil domain of TRIM28. (F) Schematic representation of segmental deletion mutants of the TRIM28 CC domain, where ΔCC1 lacks residues 246–295; ΔCC2 lacks residues 299–349. (G) MDA‐MB‐231 and MCF7 cells were co‐transfected with plasmids encoding Flag‐TRIM28 FL/ΔCC1/ΔCC2 and HA‐BRD7. Then, Co‐IP experiments were performed using an HA antibody to identify the region within the TRIM28 CC domain that binds to BRD7. (H) Based on the crystal structure of the TRIM28 RBCC region (PDB: 6QU1), two α‐helical peptides, TAB14 and TAB12, were designed to mimic the CC2 helical segment.

Given that α‐helices are crucial recognition motifs in protein‐protein interactions, we retrieved the crystal structure data of the RBCC region of TRIM28 (PDB: 6QU1) and found three α‐helices within its Coiled‐coil domain (Figure 1E). To determine which α‐helical segment binds BRD7, we constructed two TRIM28 CC domain segmental deletion mutants: ΔCC1, which lacks the first α‐helix (246–295 aa), and ΔCC2, which lacks both the second and third α‐helices (299–349 aa) (Figure 1F). Subsequent Co‐IP assays demonstrated that the TRIM28 ΔCC2 mutant completely lost its binding capacity to BRD7, whereas the ΔCC1 mutant retained full binding activity (Figure 1G). These results strongly suggest that the CC2 segment (299–349 aa) of TRIM28 serves as the structural basis for mediating the TRIM28‐BRD7 interaction, providing a precise region for designing peptides to block their interaction. Based on the α‐helical conformation of the TRIM28 CC2 region, we designed and synthesized two α‐helical peptides, TAB14 (sequence: LNKRGRVLVNDAQK) and TAB12 (sequence: IQKHQEHILRFA) (Figure 1H and Figure S2A,B). These two peptides are intended to be used for subsequent screening of anti‐tumor peptides and related mechanism studies.

### 2.2 Functional Identification of Peptides Targeting and Stabilizing the BRD7 Protein

We determined the half maximal inhibitory concentration (IC_50_) of TAB14 and TAB12 in MDA‐MB‐231, MCF7, CNE2, and Hep3B cells. As shown in Figure S3, TAB14 showed modest cytotoxicity with an IC_50_ of approximately 230 µm, while TAB12 exhibited stronger anti‐tumor activity with an IC_50_ of approximately 130 µm. To identify peptides with the specific capacity to disrupt the TRIM28‐BRD7 protein interaction, we first investigated the effects of two candidate peptides, TAB12 and TAB14, on BRD7 protein levels. MDA‐MB‐231, MCF7, CNE2, and Hep3B cells were treated with a range of concentrations of either TAB12 or TAB14 for 24 h to assess the peptides' effects across multiple tumor types. Western blot analysis revealed that TAB12 treatment induced a concentration‐dependent increase in BRD7 protein levels (Figure 2A and Figure S4A). In contrast, TAB14 had no significant impact on BRD7 expression (Figure 2B and Figure S4B). We next overexpressed TRIM28 in cancer cells, which led to a significant reduction in BRD7 protein levels. Notably, TAB12 treatment substantially reversed the TRIM28‐mediated decrease in BRD7 protein levels, whereas TAB14 did not exert such an effect (Figure 2C and Figure S4C). To further confirm the stabilizing effect of TAB12 on BRD7, we performed cycloheximide (CHX) chase assays. The results demonstrated that TAB12 treatment significantly prolonged the half‐life of BRD7 across all four cell lines, confirming its role in enhancing BRD7 protein stability (Figure 2D and Figure S4D). Based on these observations, we hypothesized that TAB12, a 12‐mer peptide derived from the CC2 region of TRIM28, might competitively occupy the binding interface between TRIM28 and BRD7 and disrupt the TRIM28‐BRD7 interaction. To validate this hypothesis, we performed co‐immunoprecipitation assays. The results showed that TAB12 treatment significantly reduced the binding between TRIM28 and BRD7, for both endogenous (Figure 2E and Figure S4E) and exogenous proteins (Figure S4F). In contrast, TAB14 treatment did not exhibit a notable disruptive effect. Moreover, subsequent ubiquitination assays in MDA‐MB‐231, MCF7, and CNE2 cells demonstrated that TAB12 treatment significantly decreased the ubiquitination level of the BRD7 protein, while TAB14 did not affect BRD7 ubiquitination (Figure 2F and Figure S4G).

**FIGURE 2:**
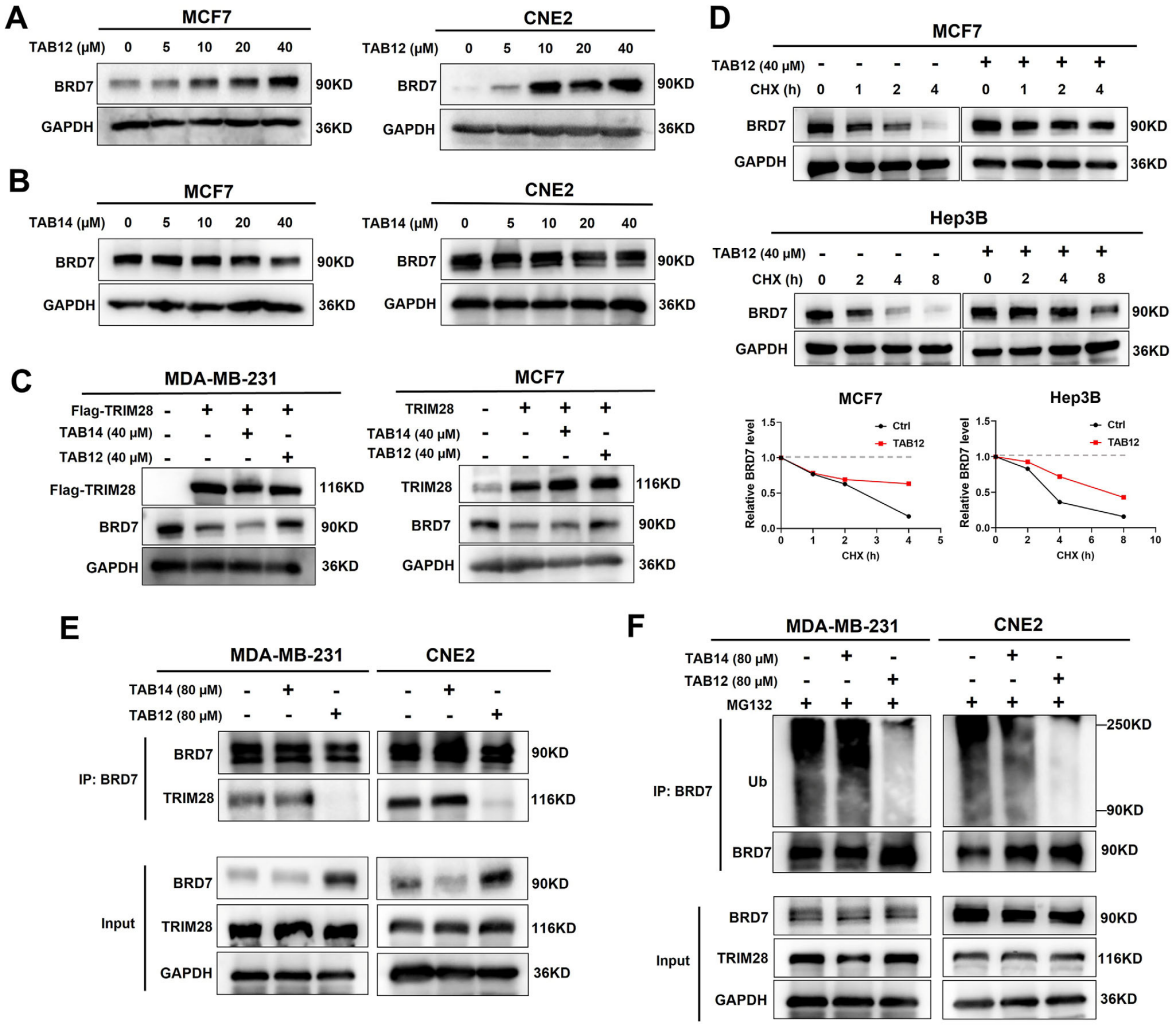
Screening and identification of the peptide TAB12 that stabilizes the BRD7 protein. (A,B) MCF7 and CNE2 cells were treated with (A) TAB12 or (B) TAB14 at gradient concentrations (0, 5, 10, 20, 40 µm) for 24 h. BRD7 protein levels were detected by Western blotting, with GAPDH as the loading control. (C) The effect of 40 µm TAB14 or TAB12 treatment for 24 h on BRD7 expression was assessed by Western blot analysis in MDA‐MB‐231 and MCF7 cells overexpressing TRIM28. (D) Following 24 h of treatment with 40 µm TAB12, cells were incubated with cycloheximide (CHX, 50 µm) to inhibit de novo protein synthesis. BRD7 protein levels at different time points after CHX addition were detected by Western blotting and quantified to generate the BRD7 degradation curve. (E) MDA‐MB‐231 and CNE2 cells were treated with 80 µm TAB14 or TAB12 for 24 h and then harvested. Co‐immunoprecipitation assays were then performed using a BRD7 antibody to analyze the effects of peptides TAB14 and TAB12 on the TRIM28‐BRD7 protein interaction. (F) MDA‐MB‐231 and CNE2 cells were treated with 80 µm TAB14 or TAB12 for 24 h, with 20 µm MG132 added for 4 h. Cell lysates were immunoprecipitated with an anti‐BRD7 antibody, and BRD7 ubiquitination was analyzed by Western blotting.

To further define the selectivity of TAB12, we assessed its impact on TRIM25, another reported E3 ligase for BRD7 [31], using Co‐IP and ubiquitination assays. Similar to its effect on TRIM28, TAB12 treatment effectively disrupted the TRIM25‐BRD7 interaction and markedly attenuated TRIM25‐mediated BRD7 ubiquitination (Figure S5A,B), demonstrating that TAB12 shields BRD7 from more than one E3 ligase. These findings suggest that the binding sites of TRIM28 and TRIM25 on BRD7 may overlap, with TAB12 competing with both ligases by occupying a shared interface. The precise mechanism warrants further investigation. We further examined whether TAB12 selectively disrupts the TRIM28‐BRD7 interaction or broadly interferes with TRIM28‐substrate binding, such as Rb, p53, or p27 [32]. As shown in Figure S5C, TAB12 specifically disrupted the TRIM28‐BRD7 interaction without affecting TRIM28 binding to Rb, p53, or p27, demonstrating that TAB12 selectively targets the TRIM28‐BRD7 interface without globally impairing TRIM28 interactions. Collectively, these results indicate that TAB12 enhances BRD7 protein stability mainly by blocking the TRIM28‐BRD7 interaction and TRIM28‐mediated ubiquitination, and it also exerts a similar inhibitory effect on the TRIM25‐BRD7 axis to further support BRD7 stabilization.

### 2.3 Peptide TAB12 Selectively Inhibits Tumor Cell Proliferation and Induces Apoptosis In Vitro

To evaluate the anti‐tumor efficacy of the candidate peptides designed to target and stabilize BRD7, we first assessed the impact of TAB14 and TAB12 on cell viability using the CCK‐8 and colony formation assays. The results revealed that treatment with TAB12 significantly suppressed the proliferation of MDA‐MB‐231, MCF7, CNE2, and Hep3B cells. In contrast, TAB14 exhibited a relatively weak inhibitory effect on these tumor cell lines (Figure 3A,B). To further determine the selective toxicity of TAB12, cancer cell lines and their corresponding normal cell lines were treated with a concentration gradient of the peptide TAB12. The data demonstrated that TAB12 specifically reduced the viability of MDA‑MB‑231, MCF7, and CNE2 in a dose‐dependent manner, but had a limited impact on the viability of normal epithelial MCF10A and NP69 cells (Figure 3C), strongly indicating its selective inhibitory activity toward malignant cells. Regarding the safety profile of TAB12, even at a concentration as high as 320 µm, it did not induce significant hemolysis in primary human red blood cells (Figure 3D and Figure S6A). These results indicate that TAB12 exhibits low hemolytic toxicity under the tested conditions, which represents a favorable property for this peptide candidate. Through Western blotting, we found that cleavage of PARP, a hallmark event of the apoptotic process, occurred in MCF7 cells treated with TAB12, whereas minimal cleaved PARP was observed in normal MCF10A cells (Figure S6B). Furthermore, we treated MDA‐MB‐231 and MCF10A cells with TAB12 for different treatment durations and then quantitatively analyzed the level of apoptosis using flow cytometry. As shown in Figure 3E, TAB12 significantly induced apoptosis in MDA‐MB‐231 cells in a time‐dependent manner. Under the same conditions, however, MCF10A cells did not exhibit a significant increase in apoptosis. Taken together, peptide TAB12 can effectively inhibit the proliferation of tumor cells and induce apoptosis in vitro, while showing low toxicity to normal cells. These findings highlight the favorable anti‐tumor selectivity and therapeutic potential of TAB12.

**FIGURE 3:**
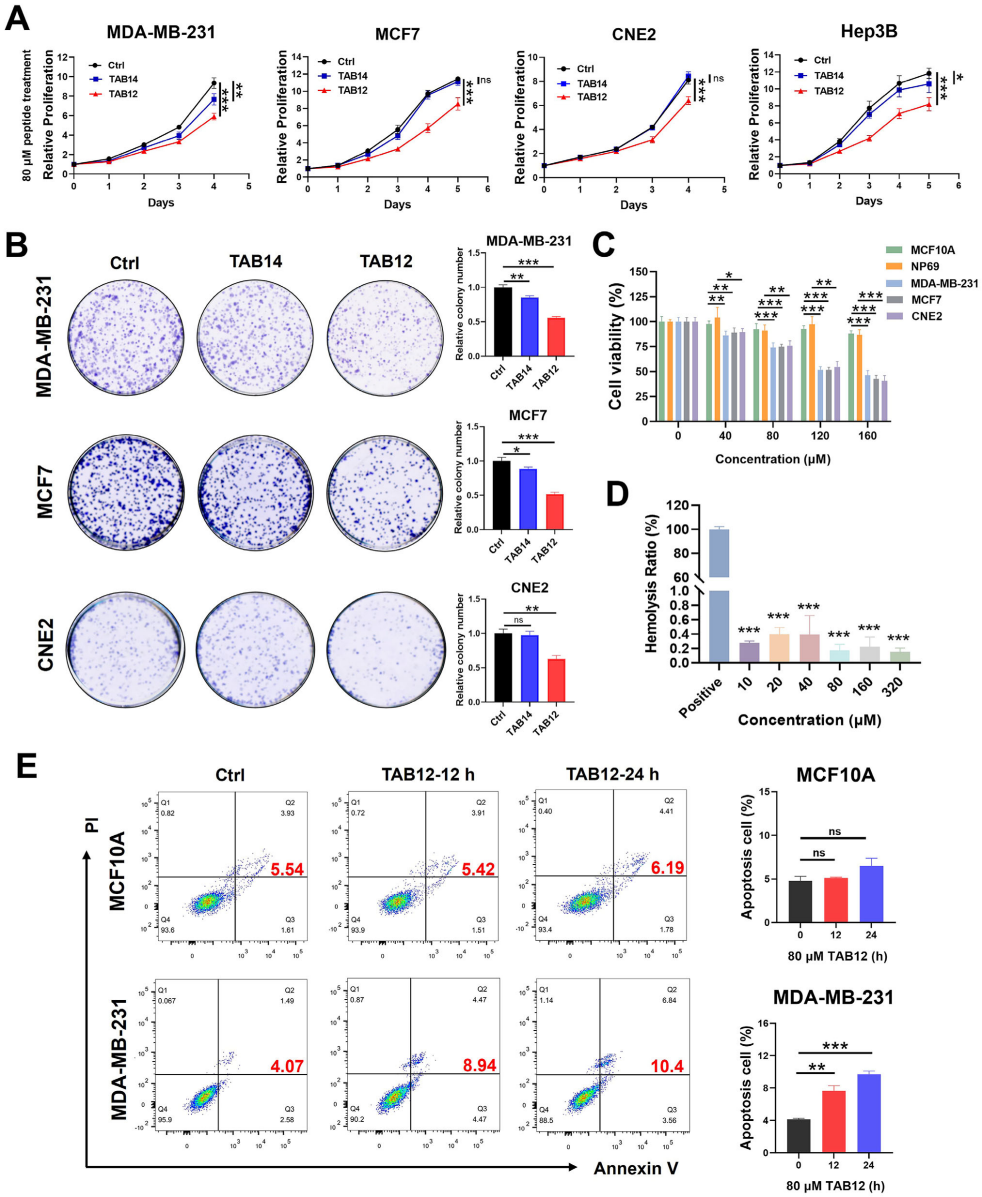
The in vitro anti‐tumor effects of the peptide TAB12. (A,B) The impacts of 80 µm peptides TAB14 and TAB12 on the proliferation and colony formation of multiple tumor cell lines were assessed via (A) CCK‐8 assay (*n* = 5, five replicates per group) and (B) colony formation assay (*n* = 3), with saline serving as the vehicle control. (C) Normal epithelial cells (MCF10A and NP69) and tumor cells (MDA‐MB‐231, MCF7, and CNE2) were incubated with TAB12 peptide at concentrations ranging from 0 to 160 µm for 24 h, and cell viability was determined using the CCK‐8 assay (*n* = 5). (D) A hemolysis assay was conducted using primary human red blood cells to evaluate the hemolytic toxicity of TAB12 at concentrations ranging from 0 to 320 µm at 37°C for 2 h (*n* = 3). (E) MCF10A and MDA‐MB‐231 cells were collected after being treated with 80 µm TAB12 for 12 or 24 h, and the levels of apoptosis were detected by flow cytometry (*n* = 3). Apoptotic cells were defined as Annexin V^+^/PI^−^ (early apoptosis) and Annexin V^+^/PI^+^ (late apoptosis) populations. The data in (A,B,C,D,E) are presented as mean ± SD (*n* ≥ 3). *, *p* < 0.05; **, *p* < 0.01; ***, *p* < 0.001; ns, no significance.

### 2.4 TAT Modification Enhances Intracellular Delivery and Anti‐Tumor Activity of TAB12

To enhance intracellular delivery, TAB12 was fused with the cell‐penetrating peptide TAT (sequence: GRKKRRQRRRPPQ) to generate TAT‐TAB12 (Figure S2C,D). TAB12 and TAT‐TAB12 were labeled with FITC to evaluate the effect of TAT modification on cellular uptake. Fluorescence cellular uptake assays (Figure 4A and Figure S7A) showed that TAB12 possesses a certain degree of cell‐penetrating ability. TAT modification significantly enhanced TAB12 uptake by tumor cells and promoted its nuclear translocation. Serum stability assays showed that unmodified TAB12 had a serum half‐life of approximately 12 h, which was not significantly altered by TAT modification (Figure 4B). These findings confirm that TAB12 inherently maintains favorable serum stability, and the TAT modification enhances cell membrane permeability without compromising this property. We then evaluated the in vitro anti‐tumor activity of TAT‐TAB12 in MDA‐MB‐231, MCF7, CNE2, and Hep3B cells using CCK‐8 and colony formation assays. TAT‐TAB12 showed stronger inhibition of tumor cell proliferation compared to unmodified TAB12, while TAT alone had no significant effect on cell viability (Figure 4C,D and Figure S7B,C). Wound healing and transwell assays revealed that, compared to TAT, TAT‐TAB12‐treated cells showed significantly slower wound healing rates (Figure 4E and Figure S7D) and fewer migrated cells passing through transwell chambers (Figure 4F and Figure S7E). These findings indicate that the peptide TAT‐TAB12, which targets the TRIM28‐BRD7 interaction, significantly inhibits the proliferation, migration, and invasion capabilities of tumor cells. To further explore the underlying mechanism, the effects of TAT‐TAB12 on cell cycle‐related and epithelial‐mesenchymal transition (EMT)‐related proteins were examined. Western blot results showed that, compared with TAT, TAT‐TAB12 increased the expression of the epithelial marker ZO‐1, while suppressing the mesenchymal markers N‐cadherin and Vimentin, as well as the cell cycle‐related molecules CDK1 and CDK4 (Figure 4G and Figure S7F). In summary, TAT modification significantly enhances the intracellular delivery efficiency of TAB12, thereby potentiating its anti‐tumor activity. The underlying mechanism may involve the regulation of cell cycle‐related and EMT‐related protein expression.

**FIGURE 4:**
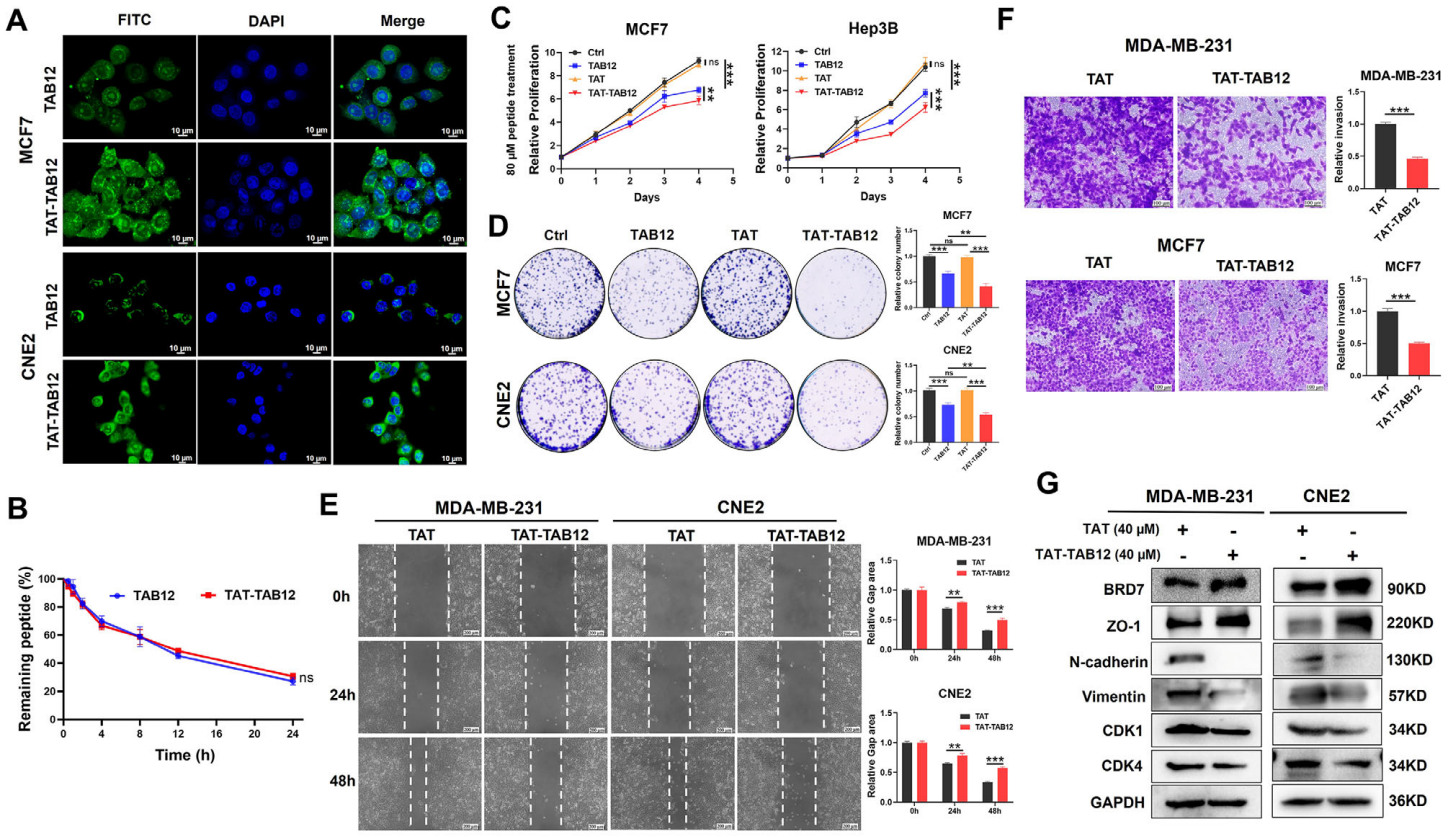
The cell‐penetrating peptide TAT enhances the intracellular delivery and anti‐tumor activity of TAB12. (A) Representative images of the cellular uptake assay. MCF7 and CNE2 cells were incubated with 30 µm FITC‐labeled TAB12 or TAT‐TAB12 peptide for 2 h. Cellular uptake of TAB12 and TAT‐TAB12 was observed by fluorescence microscopy. Cell nuclei were stained with DAPI. Scale bar, 10 µm. (B) In vitro serum stability of TAB12 and TAT‐TAB12 over time. (C) The impact of 80 µm TAB12 or its TAT‐modified peptide TAT‐TAB12 on cell proliferation was assessed using the CCK‐8 assay (*n* = 5). (D) The influence of 80 µm TAB12 or TAT‐TAB12 treatment on colony‐forming ability was examined using the colony formation assay (*n* = 3). (E) The effects of 80 µm TAT or TAT‐TAB12 on cell migration were analyzed using the wound‐healing assay (*n* = 3). (F) The influence of 80 µm TAT or TAT‐TAB12 treatment on cell invasive ability was detected by the transwell assay (*n* = 3). (G) In cells treated with 40 µm TAT or TAT‐TAB12, the expression levels of BRD7, ZO‐1, N‐cadherin, Vimentin, CDK1, and CDK4 proteins were detected by Western blot. The data in (B,C,D,E,F) are presented as mean ± SD (*n* ≥ 3). **, *p* < 0.01; ***, *p* < 0.001; ns, no significance.

### 2.5 TAB12 Enhances BRD7 Protein Stability by Competitively Inhibiting the Binding of TRIM28 to BRD7

To verify the binding of TAB12 to BRD7, we employed the cellular thermal shift assay (CETSA). The underlying principle of this technique is that a specific drug‐target interaction induces conformational changes in the target protein, thereby enhancing its thermal stability. In control samples, the BRD7 protein progressively degraded with increasing temperature. By contrast, TAB12 treatment shifted the melting curve rightward, indicating enhanced thermal stability of BRD7 relative to the control. Notably, TAT‐TAB12 further enhanced this effect, as substantial BRD7 protein was retained at higher temperatures compared to TAB12 alone (Figure 5A and Figure S8A). Furthermore, immunofluorescence staining showed that TAT‐TAB12 and BRD7 predominantly co‐localized in the nuclei of MDA‐MB‐231, MCF7, and CNE2 cells (Figure 5B and Figure S8B), indicating that their interaction occurs primarily in the nucleus. To further quantitatively determine the direct binding affinity between TAB12 and BRD7, surface plasmon resonance (SPR) analysis was performed. The results revealed a specific and strong binding interaction between TAB12 and BRD7 protein, with a dissociation constant (KD) of 200 nm (Figure 5C). Subsequently, total cell lysates were incubated with TAB12 peptide at various concentrations or with solvent alone, followed by Pronase E digestion. As shown in Figure 5D, BRD7 protein was markedly degraded in the solvent group, whereas TAB12 treatment significantly enhanced the resistance of BRD7 to Pronase E degradation in a dose‐dependent manner, further demonstrating that TAB12 binds to BRD7. Having established the direct interaction between TAB12 and BRD7, we further investigated the molecular mechanism underlying TAB12‐mediated stabilization of the BRD7 protein. We conducted CHX chase assays using cells overexpressing TRIM28. The results showed that TRIM28 overexpression accelerated BRD7 degradation, while TAT‐TAB12 treatment significantly reversed this process (Figure 5E and Figure S8C). Further Co‐IP experiments demonstrated that TAT‐TAB12 effectively reduced the level of BRD7 ubiquitination mediated by TRIM28 overexpression, while treatment with TAT alone did not exhibit this effect, suggesting that the fusion with the cell‐penetrating peptide does not affect the function of TAB12 (Figure 5F and Figure S8D). Next, after co‐transfecting cells with Flag‐TRIM28 and HA‐BRD7 plasmids, we treated the cells with 0, 20, 40, and 80 µm of TAT‐TAB12 and conducted Co‐IP analysis. The immunoblotting results showed that the peptide TAT‐TAB12 dose‐dependently inhibited the binding of TRIM28 to BRD7 (Figure 5G). Collectively, these results demonstrate that TAB12 directly and competitively binds to BRD7, thereby blocking its interaction with the E3 ligase TRIM28. Having established that TAB12 directly binds and stabilizes BRD7, we further investigated which downstream pathways are modulated by this stabilization effect. To this end, we performed RNA sequencing on MCF7 cells treated with control and TAT‐TAB12. A total of 213 differentially expressed genes (DEGs) were identified (Figure S9A,B). GSEA revealed that gene sets related to the complement and coagulation cascades, homologous recombination, mismatch repair, and DNA replication were significantly enriched among the genes downregulated by TAT‐TAB12 (Figure S9C). These findings suggest that TAT‐TAB12 stabilizes BRD7 to exert anti‐tumor effects by inhibiting DNA replication, impairing DNA damage repair to induce cell cycle arrest and apoptosis, and suppressing aberrant complement activation in the tumor microenvironment to alleviate tumor‐promoting inflammation.

**FIGURE 5:**
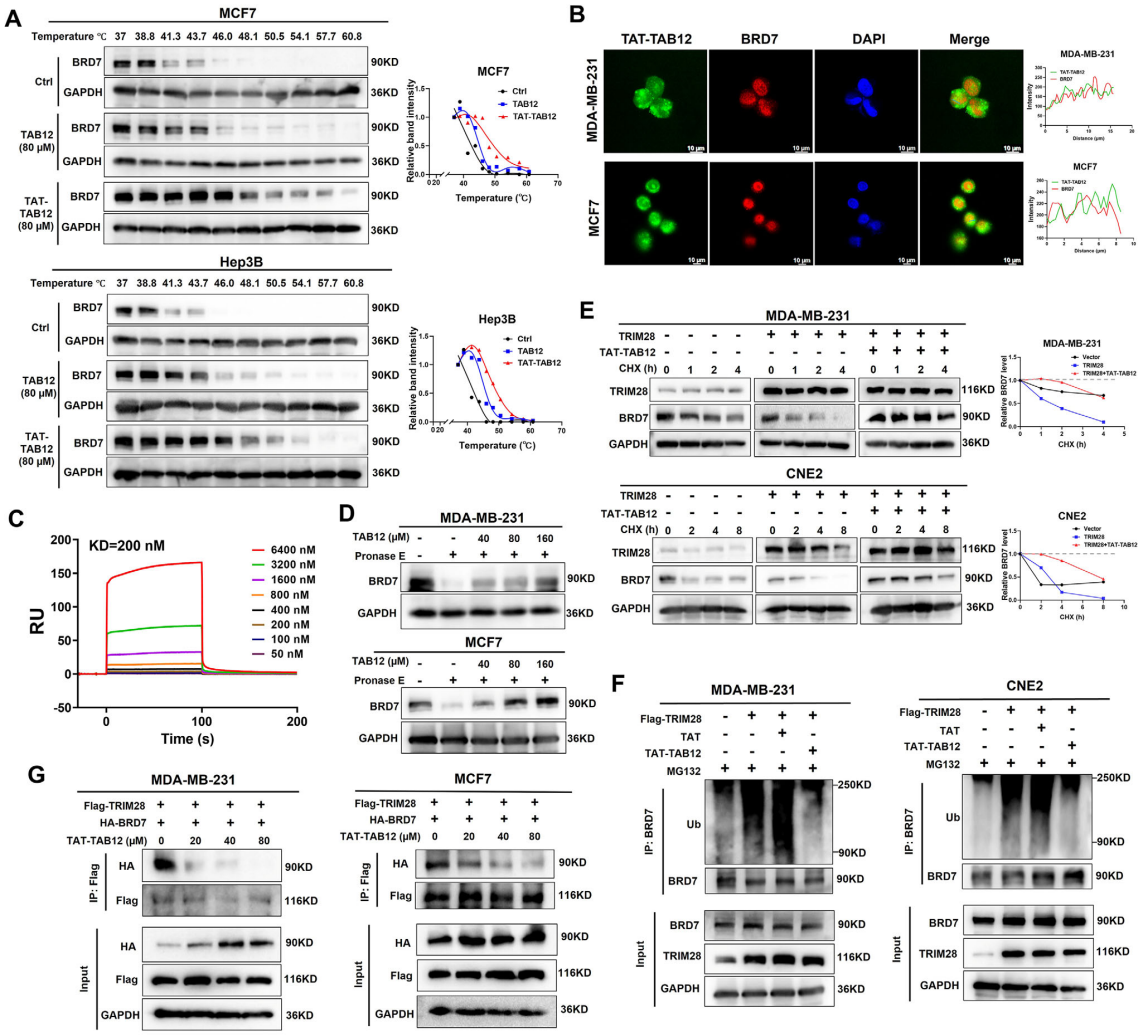
TAB12 competitively inhibits TRIM28 binding to BRD7, enhancing its protein stability. (A) Cells were incubated with 80 µm TAB12, TAT‐TAB12, or control for 12 h, followed by CETSA to evaluate BRD7 thermal stability at different temperatures, and melting curves depicting the relative protein abundance versus temperature were plotted. (B) After incubating cells with 30 µm TAT‐TAB12 for 2 h, immunofluorescence analysis was used to examine the co‐localization of TAT‐TAB12 and BRD7. TAT‐TAB12 was FITC‐labeled, endogenous BRD7 was stained with an anti‐BRD7 (red) antibody, and cell nuclei were stained with DAPI. Scale bar, 10 µm. (C) SPR assay was used to analyze the direct binding affinity between the TAB12 peptide and the BRD7 protein. (D) Protein lysates were incubated with varying concentrations of TAB12 or solvent control, followed by digestion with Pronase E diluted at 1:5000. The levels of BRD7 protein were subsequently detected by Western blot. (E) Cells overexpressing TRIM28 were treated with 40 µm TAT‐TAB12 for 12 h, then treated with CHX for different time intervals. BRD7 expression was detected by Western blot, and quantitative results are shown in a line graph. (F) After transfection with the vector or TRIM28 plasmid, cells were treated with 80 µm TAT or TAT‐TAB12 for 12 h. A ubiquitination assay was then performed to detect BRD7 ubiquitination levels. (G) Cells overexpressing Flag‐TRIM28 and HA‐BRD7 were incubated with 0, 20, 40, and 80 µm TAT‐TAB12 for 12 h. Co‐IP was then conducted to detect the binding between Flag‐TRIM28 and HA‐BRD7.

### 2.6 TAT‐TAB12 Inhibits Tumor Progression by Targeting and Stabilizing the BRD7 Protein

To determine whether the anti‐tumor effect of TAT‐TAB12 depends on its ability to stabilize BRD7, we designed a rescue experiment. BRD7 was stably knocked down in cancer cells using shBRD7, with a non‐targeting shRNA (shNC) as a control. These cells were then treated with TAT or TAT‐TAB12. Western blot analysis confirmed the efficient knockdown of BRD7 in shBRD7 cells and the stabilizing effect of TAT‐TAB12 on the BRD7 protein (Figure 6A and Figure S10A). Subsequently, we examined whether BRD7 knockdown affected cellular responses to TAT‐TAB12 using CCK‐8 and colony formation assays. The results revealed that, compared with shNC control cells, BRD7 knockdown rendered MDA‐MB‐231, MCF7, and CNE2 cells resistant to the growth inhibitory effects of TAT‐TAB12 (Figure 6B,C and Figure S10B,C). Similarly, flow cytometric analysis of apoptosis demonstrated that TAT‐TAB12 treatment induced apoptosis in MDA‐MB‐231 and MCF7 cells, and this effect was significantly attenuated upon BRD7 knockdown (Figure 6D). These data indicate that the anti‐tumor activity of TAT‐TAB12 is mediated through targeting and stabilizing BRD7. Wound healing and transwell assay results showed that BRD7 knockdown significantly attenuated the inhibitory effects of TAT‐TAB12 on cell migration and invasion (Figure 6E,F and Figure S10D). In all the above functional rescue experiments, independent shBRD7 control groups were separately set up to exclude interference from gene silencing itself, thereby precisely confirming that the effect of TAT‐TAB12 depends on BRD7. Additionally, we performed a TRIM28 overexpression rescue experiment. The results showed that TRIM28 overexpression significantly attenuated the in vitro anti‐tumor activity of TAT‐TAB12 (Figure S11A–C), suggesting that elevated TRIM28 levels may compete with TAT‐TAB12 for BRD7 binding, thereby counteracting the stabilizing effect of the peptide on BRD7 and diminishing its anti‐tumor activity. Taken together, BRD7 knockdown reversed the anti‐tumor effect of TAT‐TAB12, while TRIM28 overexpression similarly antagonized its activity, indicating that TAT‐TAB12 exerts its anti‐tumor function by targeting the TRIM28‐BRD7 interaction and stabilizing the BRD7 protein.

**FIGURE 6:**
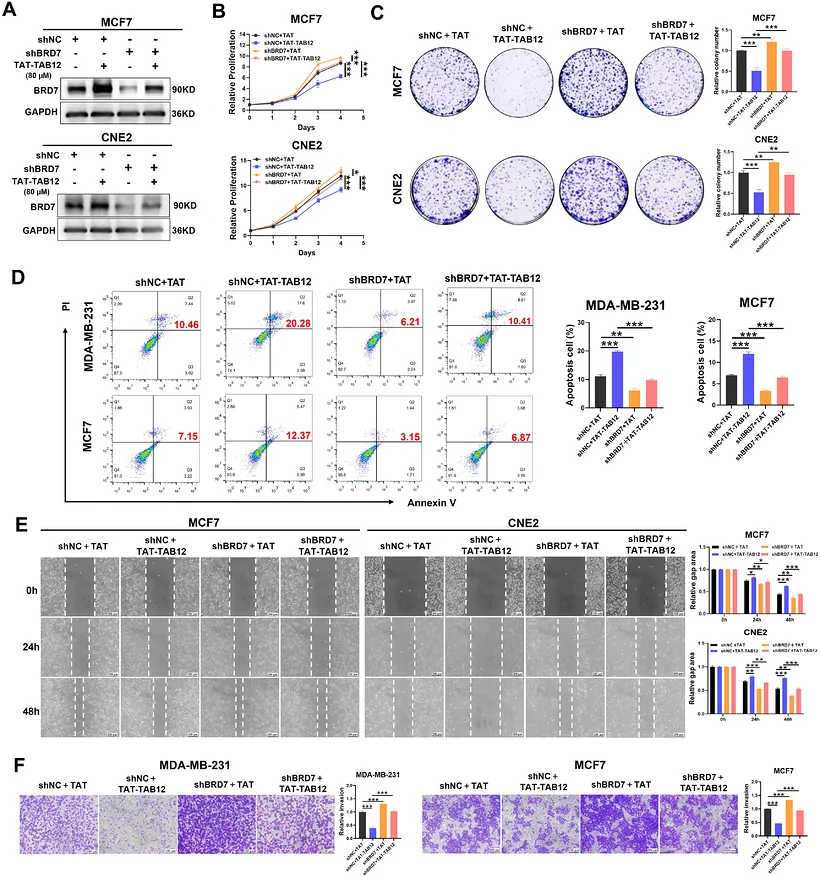
The anti‐tumor effect of TAT‐TAB12 is dependent on its targeted stabilization of the BRD7 protein. (A) BRD7 was stably knocked down in MCF7 and CNE2 cells using shBRD7, with shNC as a control. These cells were then treated with 80 µm TAT or TAT‑TAB12 for 12 h. Western blotting was performed to detect BRD7 expression levels. (B–F) Stable BRD7 knockdown (shBRD7) and control (shNC) cells were treated with 80 µm TAT or TAT‑TAB12. The effects of TAT or TAT‐TAB12 on cell proliferation, apoptosis, migration, and invasion with or without BRD7 knockdown were explored via (B) CCK‐8 assay (*n* = 5), (C) colony formation assay (*n* = 3), (D) flow cytometry analysis (*n* = 3), (E) wound healing assay (*n* = 3), and (F) Matrigel invasion assay (*n* = 3). The data in (B,C,D,E,F) are presented as mean ± SD (*n* ≥ 3). *, *p* < 0.05; **, *p* < 0.01; ***, *p* < 0.001; ns, no significance.

### 2.7 In Vivo Anti‐Tumor Efficacy and Safety Profile of TAT‐TAB12

Given the significant anti‐tumor activity of TAT‐TAB12 observed in vitro, we further evaluated its therapeutic efficacy and safety in vivo using a mouse xenograft model. Subcutaneous tumors were established in BALB/c nude mice by subcutaneously inoculating MCF7 breast cancer cells. Once tumors reached approximately 50 mm^3^, mice were randomly divided into four groups and treated with solvent (saline), TAT, TAB12, or TAT‐TAB12, respectively. Consistent with the in vitro results, TAB12 treatment significantly inhibited tumor growth and reduced final tumor weight compared to the saline group. Notably, TAT‐TAB12 exhibited superior anti‐tumor efficacy, whereas TAT alone had no significant effect on tumor growth (Figure 7A–C). These findings demonstrate the potent anti‐tumor activity of TAB12 and confirm that TAT modification effectively enhances its in vivo efficacy. Importantly, no significant body weight loss was observed across all treatment groups during the entire treatment period (Figure 7D), indicating that TAB12 and TAT‐TAB12 are well tolerated at therapeutically effective doses. Immunohistochemical staining of the xenograft tumor tissues revealed notable changes in the expression of several proteins. TAB12 treatment significantly downregulated the expression of the proliferation marker Ki67 and the mesenchymal marker Vimentin, while upregulating the expression of the tumor suppressor BRD7, the epithelial marker ZO‐1, the apoptotic marker Cleaved‐PARP, and the cell cycle inhibitor p21. Notably, in the TAT‐TAB12‐treated group, the expression changes of these molecules were more pronounced (Figure 7E). Similarly, the expression of relevant molecules in the tumor tissues was detected by Western blot. The results showed that, compared with the control groups, the expression of BRD7 and ZO‐1 was significantly upregulated in the TAT‐TAB12 treatment group, whereas the expression of Vimentin and CDK4 was significantly downregulated (Figure S12). The above results suggest that TAT‐TAB12 delays tumor progression in vivo by stabilizing the BRD7 protein, leading to suppression of cell proliferation, induction of cell cycle arrest, inhibition of EMT, and promotion of apoptosis. To systematically assess TAT‐TAB12 toxicity, we isolated the hearts, livers, spleens, lungs, and kidneys of mice for H&E staining. The results showed no obvious abnormalities or pathological changes in the tissue morphology of these organs in the TAB12 and TAT‐TAB12 treatment groups (Figure 7F). In summary, TAT‐TAB12 exhibits favorable anti‐tumor activity by stabilizing the tumor‐suppressor BRD7 and does not show significant organ toxicity.

**FIGURE 7:**
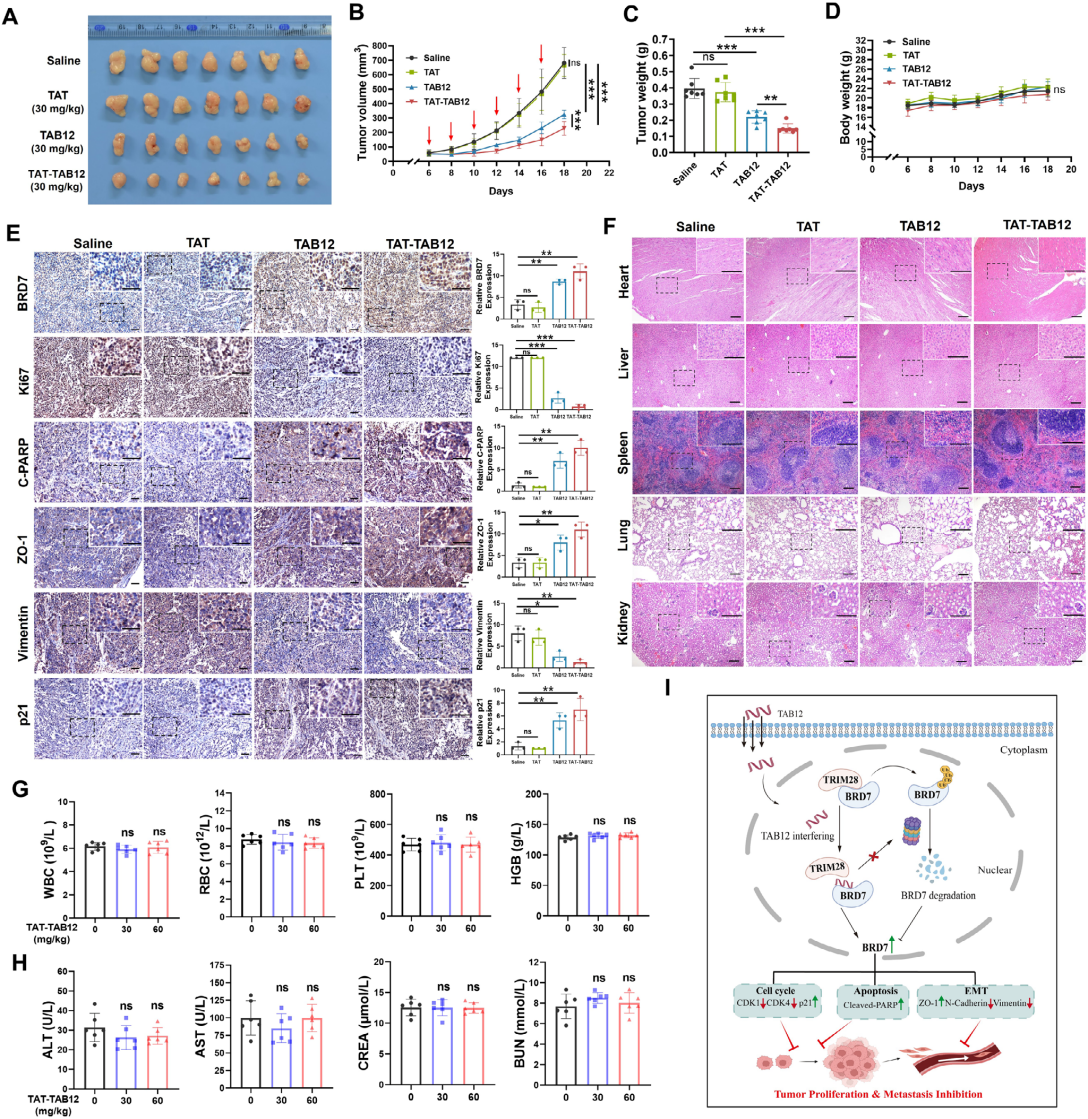
TAT‐TAB12 exhibits favorable in vivo anti‐tumor efficacy and a good safety profile. (A–F) The anti‐tumor efficacy of TAB12 and TAT‐TAB12 in a breast cancer subcutaneous xenograft model. MCF7 cells were inoculated subcutaneously into nude mice to establish xenograft tumors. Mice were treated with saline, TAT, TAB12, or TAT‐TAB12 at a dose of 30 mg/kg. (A) Image of excised xenograft tumors. (B) Growth curves of xenograft tumors (*n* = 7). (C) Statistical analysis of tumor weights (*n* = 7). (D) Changes in the body weight of mice during the treatment course (*n* = 7). (E) Representative IHC staining images and quantitative analysis in the xenograft tumor tissues of nude mice (*n* = 3). Specific region views were magnified and positioned in the upper‐right corner, with magnified areas annotated. Scale bar, 100 µm. (F) Representative images of H&E staining from the hearts, livers, spleens, lungs, and kidneys of xenograft tumor‐bearing mice. Magnified views of the selected regions are shown in the upper‐right corner, with magnified areas indicated. Scale bar, 200 µm. (G,H) Blood samples were collected from C57BL/6 mice after treatment with medium (30 mg/kg) or high (60 mg/kg) doses of TAT‐TAB12 for quantitative analysis of hematological parameters: (G) Complete blood count was performed to evaluate the hematological toxicity of TAT‐TAB12 peptide, including WBC, RBC, PLT, and HGB (*n* = 6); (H) Serum biochemical analysis was conducted to assess the hepatotoxicity and nephrotoxicity of TAT‐TAB12, covering ALT, AST, CREA, and BUN (*n* = 6). (I) Schematic diagram illustrating the molecular mechanism by which TAB12 exerts its anti‐tumor effects by blocking the interaction between TRIM28 and BRD7 proteins, thereby enhancing the stability of the BRD7 protein. The data in (B,C,D,E,G,H) are presented as mean ± SD (*n* ≥ 3). *, *p* < 0.05; **, *p* < 0.01; ***, *p* < 0.001; ns, no significance.

To further investigate the potential of TAT‐TAB12 as a safe therapeutic peptide, normal C57BL/6 mice were administered TAT‐TAB12 via tail vein injection at doses of 30 mg/kg (medium dose) or 60 mg/kg (high dose) every other day for a total of three doses. Subsequently, blood samples were collected from the mice for testing. The complete blood count results showed that, compared with the control group, medium‐ and high‐dose TAT‐TAB12 did not cause significant changes in the levels of white blood cells (WBC), red blood cells (RBC), platelets (PLT), and hemoglobin (HGB) in mice (Figure 7G). Furthermore, serum biochemical analysis indicated no significant differences in liver and kidney function markers—alanine aminotransferase (ALT), aspartate aminotransferase (AST), creatinine (CREA), and blood urea nitrogen (BUN)—among all groups (Figure 7H), suggesting that TAT‐TAB12 did not cause significant liver or kidney function impairment. In conclusion, these data demonstrate that TAT‐TAB12 is a well‐tolerated anti‐tumor peptide candidate with a favorable safety profile in vivo.

## 3 Discussion

As a crucial tumor suppressor, BRD7 is tightly regulated at the protein stability level by the E3 ubiquitin ligase TRIM28. TRIM28 specifically recognizes BRD7 and promotes its ubiquitination and subsequent proteasomal degradation, thereby inactivating the BRD7‐mediated tumor‐suppressive pathway. Consequently, the development of inhibitors that can specifically block the TRIM28‐BRD7 interaction holds promise for restoring the tumor‐suppressive function of BRD7, offering a novel strategy for tumor therapy. However, existing intervention approaches have significant limitations. Small‐molecule drugs, due to their limited size, struggle to effectively cover and bind to the relatively extensive PPI interface, resulting in limited inhibitory effects. Although biological agents, such as antibodies, are characterized by high specificity and affinity, they suffer from high molecular weights and poor membrane permeability. Moreover, these agents predominantly target extracellular or membrane surface targets, making it challenging to access the intracellular TRIM28‐BRD7 interaction interface [33]. In contrast, peptide drugs exhibit unique advantages: their molecular size is moderate, enabling them to mimic the key structural features of the PPI interface and efficiently occupy the binding domain, while also possessing a certain cell‐penetrating potential [34]. In recent years, peptides and peptidomimetics targeting protein‐binding interfaces have been continuously developed and applied. For instance, Li et al. designed a bicyclic stapled α‐helical peptide (p53‐16) targeting the p53‐MDM2/MDMX interaction interface, which effectively disrupted this interaction, reactivated the p53 pathway, and selectively suppressed tumor growth [35]. Similarly, researchers designed a stapled peptide derived from ATG16L1 based on the crystal structure of the ATG5 and ATG16L1 interaction. This stapled peptide exhibited strong binding affinity for ATG5 and could target the formation of the ATG5‐ATG16L1 complex, thereby inhibiting autophagy [36]. These examples show that designing peptides mimicking natural interaction interfaces can efficiently and selectively intervene in specific PPIs. Based on this, we precisely located the key region of the TRIM28‐BRD7 interaction and designed an α‐helical peptide, TAB12, that mimics the features of the natural binding interface. This peptide effectively blocks the recognition and degradation of BRD7 by TRIM28 through competitive binding to the BRD7 protein, ultimately achieving functional stabilization of the tumor suppressor protein BRD7. TRIM28 functions as a promiscuous E3 ubiquitin ligase capable of ubiquitinating multiple substrate proteins. Direct inhibition of its E3 ligase catalytic activity would globally perturb the homeostatic regulation of all its substrates, thereby potentially giving rise to pronounced off‐target effects and physiological dysregulation. In contrast, TAB12 selectively disrupts the TRIM28‐BRD7 interaction interface without perturbing TRIM28's interactions with other substrates, thereby exhibiting superior target specificity and reduced off‐target risk. Moreover, we observed that TAB12 disrupts the interaction between BRD7 and TRIM25, another reported E3 ubiquitin ligase, and significantly inhibits TRIM25‐mediated ubiquitination of BRD7. This may be attributed to the overlapping binding sites of TRIM28 and TRIM25 on BRD7. These findings indicate that TAB12 simultaneously blocks two distinct E3 ubiquitin ligase‐mediated degradation pathways of BRD7, thereby circumventing the compensatory proteasomal degradation that typically arises upon inhibition of a single E3 ligase, providing a critical safeguard for the effective restoration of BRD7 function.

Peptides are emerging therapeutics that inhibit tumor progression through diverse mechanisms. Many conventional anti‐tumor peptides act primarily as direct cytolytic agents. For instance, ChMAP‐28 rapidly permeabilizes and damages the cell membrane, causing necrotic cell death [37]. Other peptides, such as Lfcin B, can penetrate cells and directly initiate apoptosis through mitochondrial pathways involving caspase activation and ROS generation [38]. In addition, some anti‐tumor peptides exert effects by inhibiting angiogenesis, blocking the cell cycle, and modulating immune responses [39]. The TAB12 developed in this study does not function through membrane disruption or the engagement of broad signaling pathways. Instead, it precisely targets and stabilizes the crucial intracellular tumor suppressor BRD7, restoring its function. The resulting inhibition of proliferation, migration, and invasion, as well as the induction of apoptosis, are therefore downstream effects of BRD7 functional restoration. Moreover, these effects strictly depend on the presence of BRD7, which fully demonstrates the high targeting specificity of TAB12. This targeting strategy, centered on regulating protein stability, differs fundamentally from traditional anti‐tumor peptides. Rather than directly killing cells, it corrects protein homeostasis imbalance in tumor cells, restores intrinsic tumor‐suppressive pathways, and thus specifically inhibits tumor growth. This strategy provides a new intervention approach for tumor suppressor proteins that have long been considered “undruggable” due to the difficulty in effectively targeting PPI interfaces. It is particularly suitable for targets whose functional inactivation mainly results from abnormal degradation rather than gene mutations.

Compared with traditional small‐molecule drugs, peptide compounds offer numerous advantages, including low immunogenicity, high specificity, and high targeting efficiency for “undruggable” PPIs [40]. However, their inherent limitations cannot be overlooked. For instance, peptides often exhibit relatively weak cell membrane permeability. In the physiological environment, peptides are prone to being recognized and hydrolyzed by various enzymes, exhibiting poor stability [41]. Unlike small‐molecule drugs that directly target active sites, peptide‐derived PPI inhibitors must competitively engage large and relatively flat interaction interfaces, which often necessitates higher concentrations to achieve adequate target occupancy. To enhance the intracellular delivery of therapeutic molecules, cell‐penetrating peptides (CPPs) are frequently employed [42, 43]. For example, TAT, a transcriptional activator identified from the HIV‐1, can traverse the cell membrane and translocate into the nucleus [44]. In this study, by fusing the cell‐penetrating peptide TAT, we have preliminarily improved the uptake efficiency and anti‐tumor activity of TAB12. Importantly, TAT conjugation did not compromise its serum stability and function. Nevertheless, to advance its clinical application, it is essential to adopt advanced strategies in peptide drug development. On the one hand, the stability of peptides can be enhanced through structural modifications, such as substituting L‐amino acids with D‐amino acids, performing site‐specific modifications on side chains, and constructing stapled peptides via alkenyl bridging. On the other hand, novel delivery systems can be utilized to improve their tissue penetration and targeting ability, including liposome encapsulation, nanoparticle‐based delivery, and cell‐specific ligand modification [45].

In summary, this study developed a peptide inhibitor TAB12 targeting TRIM28‐BRD7 interaction, confirming its selective anti‐tumor effect via stabilizing BRD7. It not only offers a peptide drug with translational potential for cancer therapy but also breaks through the traditional paradigm of inhibiting the activity of oncogenic proteins, opening a new path for anti‐tumor drug development targeting challenging PPIs. Although there remain hurdles to overcome in terms of peptide druggability before clinical translation, with advances in synthesis, modification, and delivery technologies, TAB12‐like highly specific PPI inhibitor peptides promise novel precision oncology strategies.

## 4 Conclusion

This study focused on the TRIM28/BRD7 signaling axis, which plays a pivotal role in tumorigenesis and tumor progression. Based on the key structural domain mediating TRIM28‐BRD7 interaction, we designed a peptide inhibitor, TAB12, targeting this protein‐protein interaction interface. TAB12 binds to BRD7 with high affinity and specifically disrupts TRIM28 recruitment to BRD7, thereby inhibiting ubiquitin‐proteasome degradation of BRD7 and markedly increasing its protein stability. In vitro functional assays showed that TAB12 selectively suppressed the proliferation, migration, and invasion of tumor cells and induced tumor cell apoptosis. Moreover, these anti‐tumor effects are strictly dependent on its ability to stabilize BRD7, supporting the on‐target activity of TAB12. Further in vivo animal experiments have demonstrated that TAB12 can exert favorable anti‐tumor efficacy by activating the BRD7 protein, without causing obvious toxicity. Conjugation of the cell‐penetrating peptide TAT improved the cellular uptake and anti‐tumor activity of TAB12. In summary, this study reports a novel peptide‐based BRD7 stabilizer, TAB12, targeting the TRIM28‐BRD7 interaction interface. Our findings provide a promising lead compound for tumors with low BRD7 expression and a feasible strategy for targeting undruggable protein‐protein interactions in cancer therapy (Figure 7I).

## 5 Materials and Methods

### 5.1 Peptide Synthesis

All peptides (TAB14, TAB12, TAT, and TAT‐TAB12) used in this study were synthesized by DankPeptide using Fmoc Solid Phase Peptide Synthesis (Fmoc‐SPPS). The crude products were then purified using reversed‐phase high‐performance liquid chromatography (RP‐HPLC) to obtain peptides with >95% purity. The resulting powders were stored at −20°C. For experiments, the peptides were dissolved in sterile saline to prepare 10 mm stock solutions for subsequent use.

### 5.2 Cell Culture

The breast cancer (MDA‐MB‐231, MCF7) and normal mammary epithelial (MCF10A) cell lines were obtained from ATCC. The normal nasopharyngeal epithelial (NP69), nasopharyngeal carcinoma (CNE2), and hepatocellular carcinoma (Hep3B) cell lines were purchased from the Cell Center of Central South University (Changsha, China). MDA‐MB‐231, MCF7, and Hep3B cells were grown in DMEM (VivaCell, C3103), while CNE2 cells were cultured in RPMI‐1640 medium (VivaCell, C3001). Both media were supplemented with 10% fetal bovine serum (FBS, Gibco, A5256701) and 1% Penicillin‐Streptomycin (PS, NCM, C100C5). NP69 and MCF10A cells were cultured in their corresponding specialized complete media (Procell, CM‐0804, CM‐0525). All cell lines were cultured at 37°C in a 5% CO_2_ atmosphere.

### 5.3 Cell Transfection

Cells were seeded into a six‐well culture plate. Following 12 h of incubation, when the cell confluence reached approximately 60%–70%, transfection was carried out. The plasmid was transfected into the cells using Polyplus transfection reagent (jetPRIME, 101000046) in accordance with the manufacturer's protocol.

### 5.4 Western Blot

After centrifugation, cell pellets were lysed in RIPA buffer with protease inhibitor cocktail, and protein concentrations were determined by BCA assay. Equal amounts of denatured proteins were separated by 10% SDS‐PAGE and transferred onto PVDF membranes. Membranes were blocked with 5% non‐fat milk, incubated overnight at 4°C with primary antibodies, washed with 1×TBST, and probed with HRP‐conjugated secondary antibody. Signals were detected using an ECL substrate and a chemiluminescence imaging system. Primary antibodies: BRD7 (Proteintech, #51009‐2‐AP, 1:3000); TRIM28 (Proteintech, #15202‐1‐AP, 1:3000); GAPDH (Proteintech, #10494‐1‐AP, 1:10000); Flag (Sigma, #F2555, 1:500); HA (MBL, #M132‐3, 1:1000); Ub (Santa Cruz, #sc‐8017, 1:1000); ZO‐1 (Proteintech, #21773‐1‐AP, 1:1000); Vimentin (Abcam, #EPR3776, 1:1000); N‐cadherin (Proteintech, #22018‐1‐AP, 1:1000); CDK1 (Abclonal, #A11420, 1:1000); CDK4 (Abclonal, #A23522, 1:1000); TRIM25 (Abclonal, #A12938, 1:2000); Rb (Abclonal, #A28031, 1:5000); p53 (Proteintech, #10442‐1‐AP, 1:2000); p27 (Affinity, #AF6324, 1:1000); Total‐PARP (Affinity, #DF7198, 1:1000); and Cleaved‐PARP (Affinity, #AF7023, 1:1000).

### 5.5 Co‐Immunoprecipitation

Cells from 10‐cm dishes were harvested and lysed in 1 mL of IP lysis buffer with cocktail. An aliquot of the lysate was saved as input. Magnetic beads were incubated with the antibodies in IP lysis buffer for 2 h at room temperature to generate antibody‐bead conjugates. Subsequently, 1 mg of protein sample was added to the IP and IgG groups, followed by an overnight incubation at 4°C. Beads were collected, washed five times with cold high‑salt buffer, and then boiled in IP lysis buffer containing loading buffer to denature bound proteins. Finally, the input, IgG control, and IP sample were analyzed by Western blot.

### 5.6 Ubiquitination Assay

For ubiquitination assays, cells were treated with 20 µm MG132 for 4 h, harvested, and lysed in a denaturing lysis buffer containing 1% SDS. Lysates were boiled at 100°C for 10 min to disrupt non‐covalent protein interactions. The lysates were subjected to immunoprecipitation with BRD7 antibody‐conjugated beads overnight at 4°C. Beads were washed five times with high‑salt washing buffer, and the immunoprecipitates were analyzed by Western blotting with an anti‐ubiquitin antibody.

### 5.7 CHX Chase Assay

Following exposure to the 40 µm peptides for 24 h, cells were incubated in fresh complete medium supplemented with 50 µm cycloheximide (CHX). Cells were harvested at the indicated time points after CHX addition for protein extraction. The protein level of BRD7 was assessed by immunoblotting. Grayscale intensity of the bands was quantified using ImageJ software, and relative BRD7 protein levels were normalized to the loading control.

### 5.8 CCK‐8 and Colony Formation Assays

After counting, cells were seeded at 100 µL/well in 96‐well plates with five replicates per group. After adhesion, cells were exposed to 80 µm peptide drug or corresponding controls. After the indicated treatment times, the original medium was removed, and 100 µL of fresh complete medium containing 10% CCK‐8 was added. Cells were incubated for 2–3 h at 37°C, and the absorbance at 450 nm was measured using a microplate reader. For colony formation, cells were seeded in six‐well plates at 1500 cells per well and treated with 80 µm peptide drug for 72 h. After 14 days of culture, colonies were fixed with 4% paraformaldehyde and stained with crystal violet.

### 5.9 Wound Healing Assay

Cells were seeded into six‐well plates and cultured overnight to >95% confluence. A straight scratch wound was then created in the center of each well. After a PBS wash, serum‐free medium containing the 80 µm peptide was added. Wound regions were imaged at 0, 24, and 48 h post‐wounding, and three random fields per group were selected for quantitative analysis of the wound area.

### 5.10 Transwell Invasion Assay

5×10^4^ cells were inoculated into the upper chamber of a Matrigel‐precoated Transwell chamber. The lower chamber was filled with a high‐serum medium as a chemoattractant. After incubation with 80 µm peptides for 48 h, the chambers were rinsed twice with PBS, and non‐invaded cells in the upper chamber were removed. Invaded cells on the lower surface were fixed with paraformaldehyde for 30 min, stained with crystal violet for 15 min, and air‐dried after washing. Finally, cells in three random fields were imaged and counted.

### 5.11 Apoptosis Assay

When cell confluence reached ≥90%, the medium was replaced with low‐serum medium containing 80 µm peptide. After 48 h of treatment, both floating cells in the supernatant and adherent cells were collected, washed twice with PBS, and apoptosis was detected using an Annexin V/PI apoptosis detection kit. Briefly, cells were resuspended in 500 µL of binding buffer, stained with 5 µL Annexin V‐iFluor 488 and 5 µL PI, and incubated at room temperature in the dark for 15–20 min. Apoptosis was then analyzed using a BD flow cytometer.

### 5.12 Hemolysis Assay

Fresh human blood collected in heparin tubes was centrifuged at 2000 rpm for 10 min to discard plasma, then washed three times with normal saline and resuspended as a 4% (v/v) erythrocyte suspension. Peptides at 20, 40, 80, 160, and 320 µm were added to the erythrocyte suspension and incubated at 37°C for 2 h, with distilled water and normal saline as positive and negative controls, respectively. After incubation, the mixtures were centrifuged again, and the supernatants were transferred to a 96‐well plate. Absorbance at 540 nm was measured using a microplate reader, and the hemolysis rate was calculated.

### 5.13 Peptide Uptake

Cells were seeded on coverslips at 5 × 10^4^ cells per well and incubated at 37°C for 24 h. The medium was then replaced with fresh medium containing 30 µm FITC‐TAB12 or FITC‐TAT‐TAB12. After 2 h, cells were washed twice with PBS and fixed with 4% paraformaldehyde for 15–20 min. Nuclei were stained with DAPI for 10 min and mounted with anti‐fade mounting medium. Fluorescence was visualized using a fluorescence microscope.

### 5.14 Cell Thermal Shift Assay (CETSA)

After 12 h treatment with 80 µm peptide drug or control, cells were harvested, washed twice with PBS, and resuspended in cold PBS containing protease inhibitors. The cell suspension was aliquoted into 10 PCR tubes (100 µL each). Samples were then heated for 3 min in a PCR thermocycler using a preset temperature gradient. Cells were then lysed by three freeze‐thaw cycles in liquid nitrogen and a 37°C water bath. The lysates were centrifuged at 12 600 rpm for 30 min at 4°C. Supernatants were collected, mixed with 5 × loading buffer, and boiled. BRD7 levels were detected by Western blotting.

### 5.15 Immunofluorescence Colocalization Assay

Cells were seeded on coverslips and cultured to 30%–40% confluence, followed by incubation with 30 µm FITC‐labeled TAT‐TAB12 for 2 h. Cells were fixed with 4% paraformaldehyde for 15 min, permeabilized with 0.3% Triton X‐100 for 10 min, and blocked with 5% goat serum for 1 h. Cells were then incubated with anti‐BRD7 antibody (1:200) overnight at 4°C, followed by Alexa Fluor 594‐conjugated secondary antibody (1:500) for 1 h at room temperature. Nuclei were counterstained with DAPI, and coverslips were mounted with an anti‐fade mounting medium. Colocalization of FITC‐TAT‐TAB12 (green) and BRD7 (red) was visualized by confocal microscopy.

### 5.16 Drug Affinity Responsive Target Stability (DARTS)

Total cellular proteins were extracted using a non‐denaturing lysis buffer without protease inhibitors and aliquoted at 250 µg per tube. TAB12 peptide was added to the lysates at final concentrations of 0, 40, 80, or 160 µm and incubated for 1 h. Pronase E was then added to lysates at a pronase‐to‐protein ratio of 1:5000, and digestion was carried out at 37°C for 30 min. Reactions were terminated with PMSF and immediately analyzed by Western blotting.

### 5.17 Surface Plasmon Resonance (SPR) Assay

The binding affinity between TAB12 and BRD7 was determined by SPR using a Biacore 1K instrument. Recombinant BRD7 protein was immobilized on a CM5 sensor chip via amine coupling. Gradient concentrations of TAB12 were then injected sequentially with an association time of 100 s and a dissociation time of 120 s, during which real‐time binding and dissociation were monitored. The dissociation constant (KD) was calculated using Biacore 1K evaluation software.

### 5.18 Serum Stability Assay

The in vitro stability of TAB12 and TAT‐TAB12 was evaluated in fresh human serum. Peptides were dissolved in sterile PBS (1 mg/mL) and diluted 1:9 in human serum to a final concentration of 100 µg/mL, followed by incubation at 37°C. Samples were collected at 0, 0.5, 1, 2, 4, 8, 12, and 24 h, followed by protein precipitation with pre‐cooled acetonitrile. After incubation at 4°C for 30 min, the mixtures were centrifuged at 20 000 rpm for 15 min, and the supernatants were collected for quantification of the remaining intact peptide.

### 5.19 Therapeutic Effect

Female BALB/c nude mice (4 weeks, 17 ± 1 g) used in this study were purchased from Hunan SJA Laboratory Animal Co., Ltd. MCF7 cells (3.0 × 10^6^ cells/100 µL) were mixed with Matrigel and then subcutaneously implanted into the right axillary region of the nude mice. When tumor volumes reached approximately 50 mm^3^, the mice were randomly assigned to four groups (*n* = 7). Subsequently, the mice received intratumoral injections of either TAB12 or TAT‐TAB12 at a dose of 30 mg/kg every other day, with saline and TAT serving as controls. Tumor volumes and body weights were monitored regularly. Tumor volume was calculated using the formula: Tumor volume = (width^2^ × length)/2. At the endpoint, mice were euthanized, and tumors were excised and weighed. Tumor tissues were collected for further analysis.

### 5.20 In Vivo Safety Evaluation

To evaluate the systemic safety of the peptide drug, 8‐week‐old C57BL/6 mice received tail vein injections of TAT‐TAB12 at 30 or 60 mg/kg every other day for a total of three doses. Twenty‐four hours after the final injection, mice were euthanized, and blood samples were collected for complete blood count analysis and serum liver and kidney function tests.

### 5.21 Immunohistochemistry (IHC)

Tissues were fixed in 4% paraformaldehyde overnight, embedded in paraffin, and sectioned. The slides were then baked, deparaffinized in xylene, and rehydrated through a series of ethanol solutions. Antigen retrieval was performed by heating in sodium citrate buffer (pH 6.0) for 20 min. After blocking endogenous peroxidase activity, the sections were incubated with a specific primary antibody, followed by biotin‐labeled goat anti‐mouse/rabbit IgG secondary antibody. Signal was visualized using DAB, and hematoxylin was used as a counterstain. Primary antibodies used: BRD7 (Proteintech, #51009‐2‐AP, 1:300), Ki67 (Santa Cruz, #sc‐23900, 1:500), Cleaved‐PARP (Affinity, #AF7023, 1:300), ZO‐1 (Proteintech, #21773‐1‐AP, 1:500), Vimentin (Proteintech, #10366‐1‐AP, 1:500), and p21 (CST, #2947, 1:500). IHC scores were calculated by multiplying staining intensity (0, negative; 1, weak; 2, moderate; 3, strong) by the percentage of positive cells (1, ≤25%; 2, 26%–49%; 3, 50%–74%; 4, ≥75%). Scores >6 were defined as high expression.

### 5.22 Hematoxylin‐Eosin (H&E) Staining

Sections were baked at 65°C for 2–3 h, deparaffinized in xylene, and rehydrated through a descending ethanol series. Sections were stained with hematoxylin for 5 min, rinsed in running tap water, differentiated in 1% hydrochloric acid‐ethanol for 5–15 s, rinsed, and blued in 1% ammonia water. Subsequently, sections were counterstained with eosin for 1–2 min. After rinsing, sections were dehydrated through an ascending ethanol series and cleared in xylene. Finally, sections were mounted with neutral balsam and imaged under a light microscope.

### 5.23 Statistical Analysis

All data are presented as mean ± SD from at least three independent experiments. Differences between two groups were assessed using Student's *t*‐test, and comparisons among multiple groups were performed using one‐way ANOVA. All data analyses were performed using GraphPad Prism 8.0. *p* value < 0.05 was considered statistically significant (ns, *p* > 0.05; *, *p* < 0.05; **, *p* < 0.01; ***, *p* < 0.001).

## Author Contributions

M.Z. and Q.W. conceived and designed the study and drafted and revised the manuscript. Q.W. and C.X. primarily designed and performed the experiments and interpreted the data. M.L. and J.W. provided technical guidance and participated in some experiments. L.Z., S.C., and Y.W. assisted in solution preparation and reagent ordering. Y.D. performed the H&E staining of nude mouse tissue sections. W.X. and Y.L. provided critical comments on the manuscript. All authors read and approved the final manuscript.

## Funding

This study was supported by grants from the National Natural Science Foundation of China (grant no. 82473262), the Key Project of Hunan Provincial Natural Science Foundation (2026JJ30026), the Hunan Provincial Key Research and Development Program (2023SK2008), and the Program of Introducing Talents of Discipline to Universities (grant no. 111‐2‐12).

## Ethics Approval and Consent to Participate

This study was approved by the Ethics Review Committees/Institutional Review Boards of Central South University (Changsha, China). All animal experiments in this study were approved by the Institutional Animal Care and Use Committee (IACUC) of Central South University (Changsha, China).

## Conflicts of Interest

The authors declare no conflicts of interest.

## Supporting information




**Supporting File**: advs76974‐sup‐0001‐SuppMat.pdf.

## Data Availability

The data that supports the findings of this study are available in the supplementary material of this article.
