# Supplementary material for: TRIM28‐Derived Peptide Exerts Anti‐Tumor Roles by Stabilizing Tumor Suppressive BRD7 Protein in Multiple Cancers
Source: Adv Sci (Weinh). 2026 Aug 5;13(60):e76974. doi: 10.1002/advs.76974 (PMC13440194; doi:10.1002/advs.76974)
Supplement: Supplementary file 1 [file ADVS-13-e76974-s001.pdf]

Supporting Information

TRIM28-derived peptide exerts anti-tumor roles by stabilizing tumor suppressive BRD7 protein in multiple cancers

*Qingqing Wei[1,2,3], Changning Xue[1,2,3], Mengna Li[1,2,3], Jianxia Wei[1,2,3], Lemei Zheng[1,2,3], Shipeng Chen[1,2,3], Yijie Wang[4], Yumei Duan[2,3], Yanwei Luo[5], Wei Xiong[1,2,3], Ming Zhou[1,2,3]**

1 NHC Key Laboratory of Carcinogenesis, Hunan Key Laboratory of Oncotarget Gene, Hunan Cancer Hospital, and the Affiliated Cancer Hospital of Xiangya School of Medicine, Central South University, Changsha, China

2 Cancer Research Institute and School of Basic Medical Sciences, Central South University, Changsha, China

3 The Key Laboratory of Carcinogenesis and Cancer Invasion of the Chinese Ministry of Education, Central South University, Changsha, China

4 Xiangya School of Medicine, Central South University, Changsha, China

5 Department of Blood Transfusion, The Third Xiangya Hospital, Central South University, Changsha, Hunan, China.

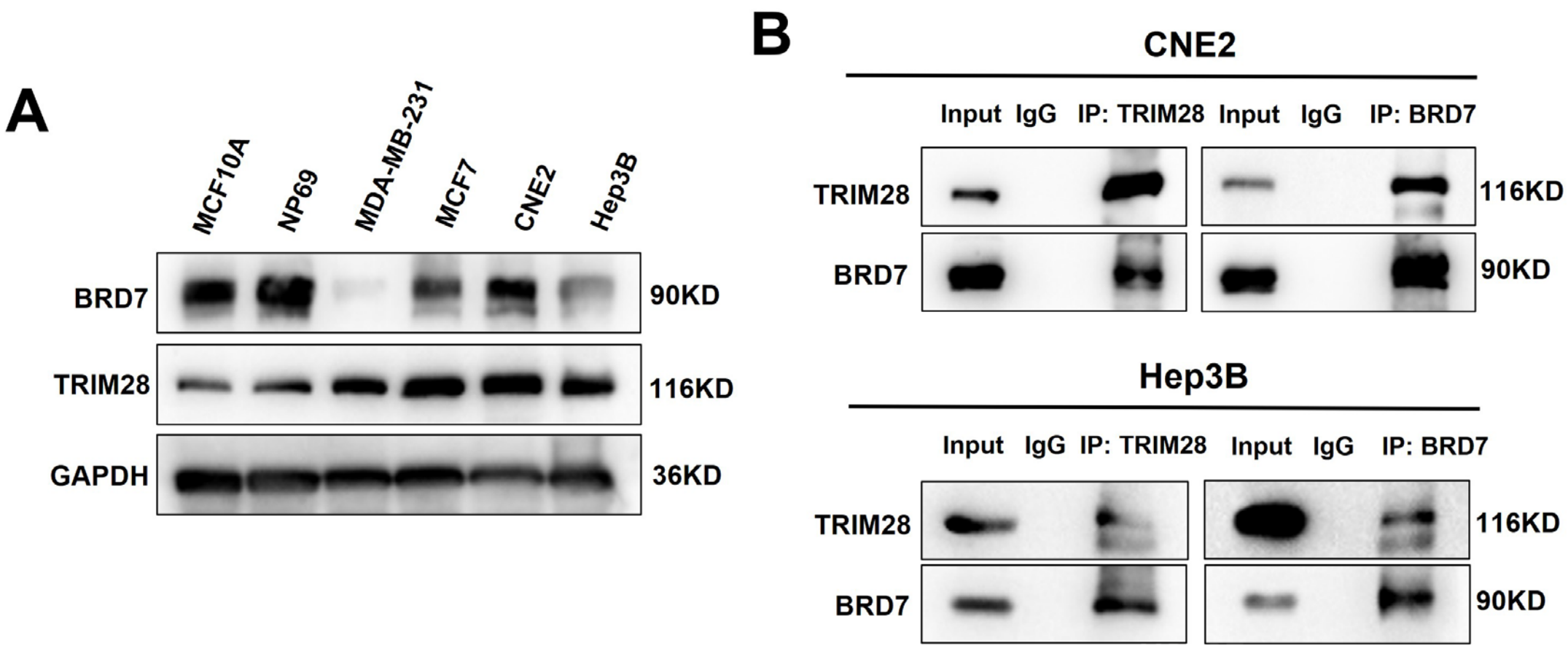

Figure S1 | TRIM28 and BRD7 interact in nasopharyngeal carcinoma and hepatocellular carcinoma cells. (A) Western blot analysis was performed to examine the protein expression levels of TRIM28 and BRD7 in a panel of normal epithelial cells, including mammary epithelial cells (MCF10A) and nasopharyngeal epithelial cells (NP69), as well as cancer cell lines, including breast cancer cells (MDA-MB-231 and MCF7), nasopharyngeal carcinoma cells (CNE2), and hepatocellular carcinoma cells (Hep3B). GAPDH was used as a loading control. (B) Co-IP assays were performed using lysates from CNE2 and Hep3B cells with antibodies against TRIM28 and BRD7 to detect the interaction between TRIM28 and BRD7. IgG was used as a negative control.

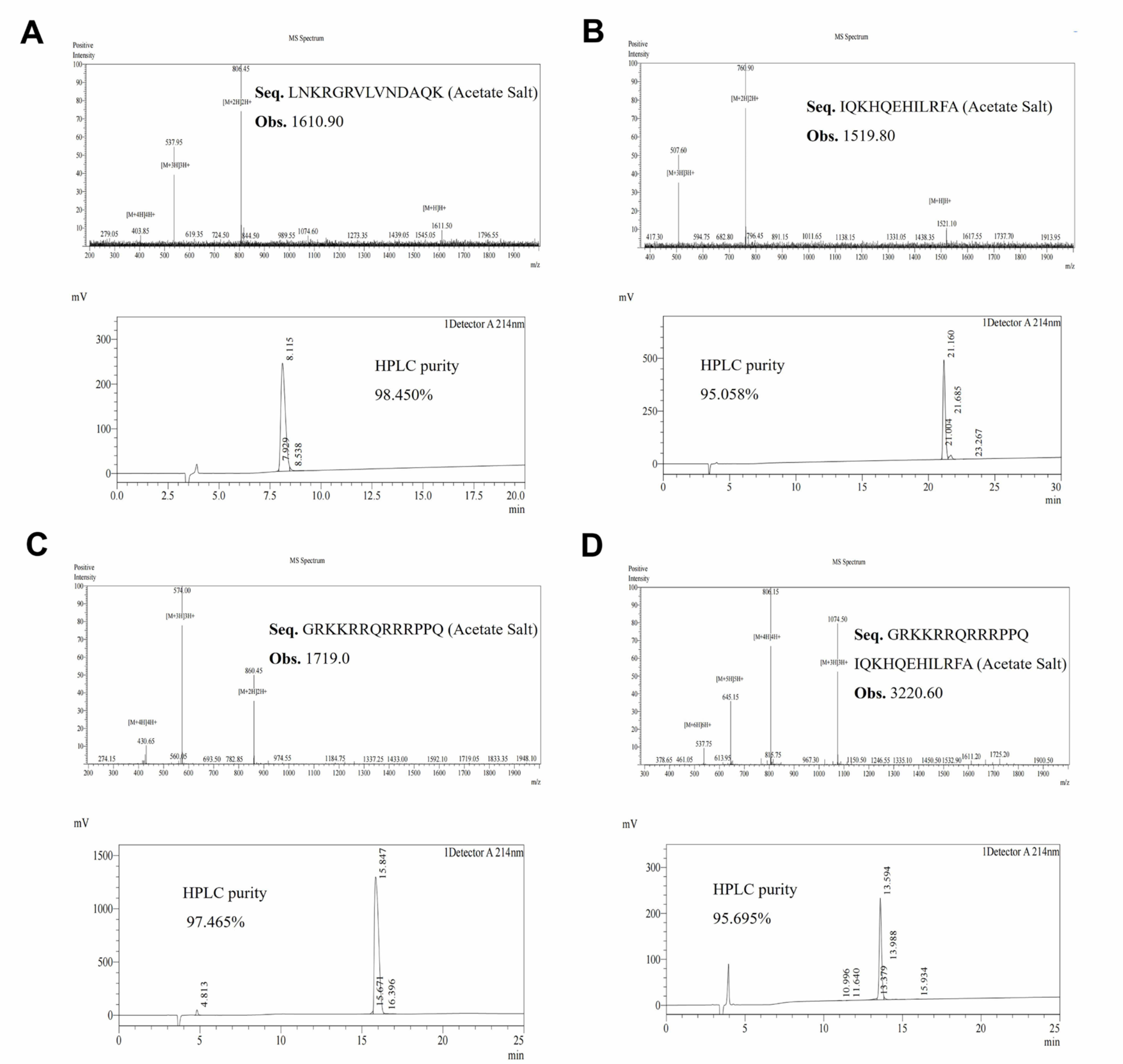

Figure S2 | Mass spectrometry and HPLC analysis of the peptides used in this study. (A) TAB14, (B) TAB12, (C) TAT, and (D) TAT-TAB12 are shown in this figure, respectively.

A

Cell line	TAB12-24h (IC_{50}, μM)	TAB14-24h (IC_{50}, μM)
MDA-MB-231	150.3	251.6
MCF7	130.3	220.8
CNE2	136.6	241.2
Hep3B	115.4	212.6

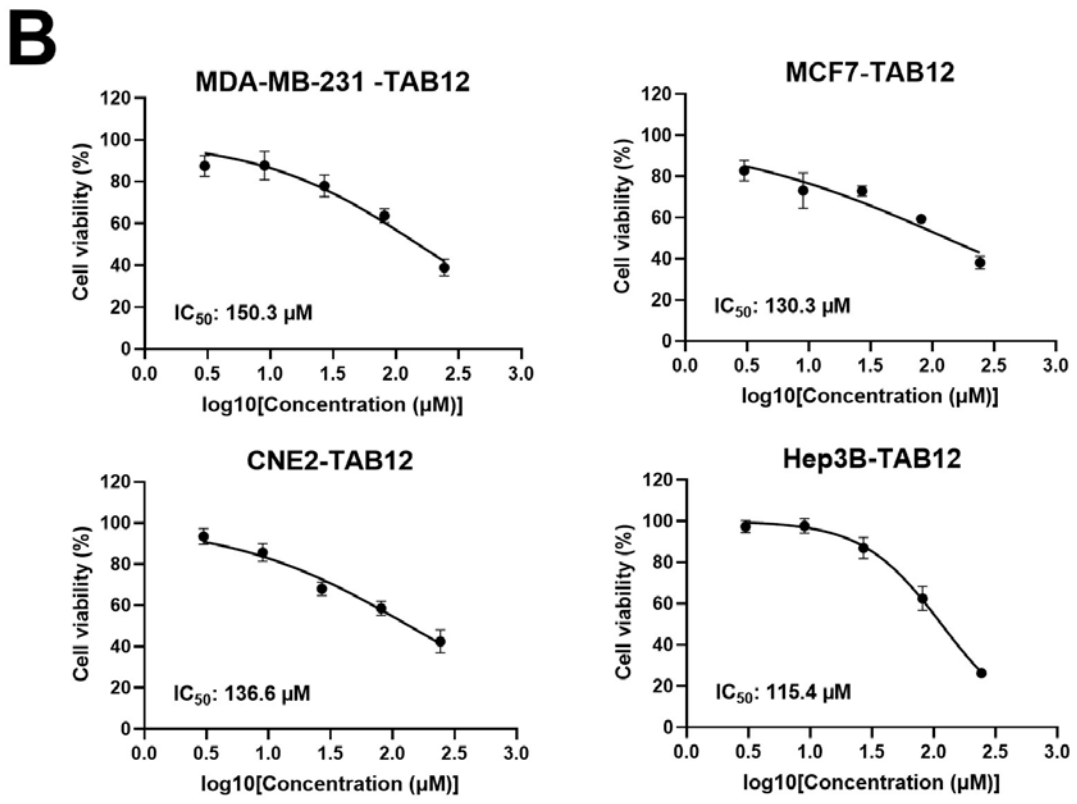

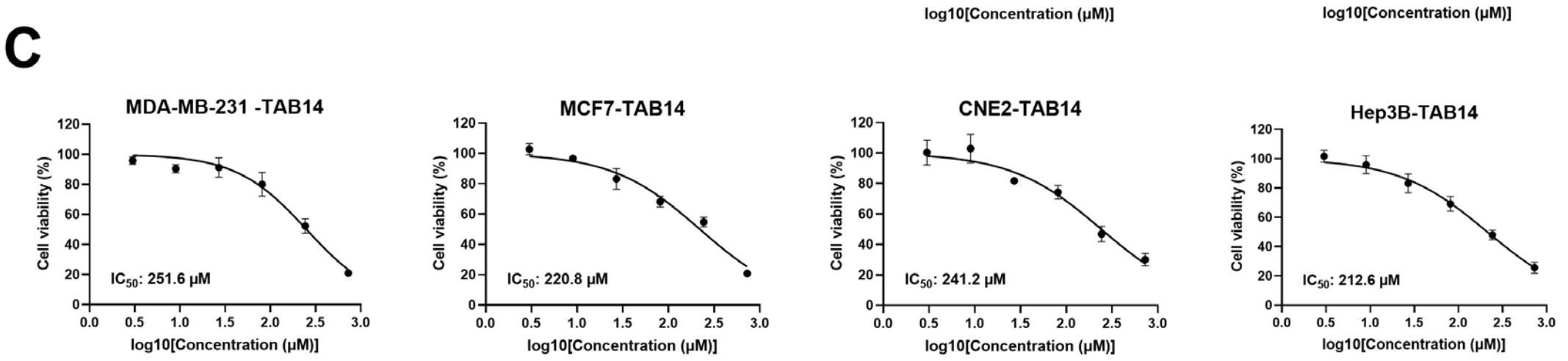

Figure S3 | IC_{50} values of peptides in multiple cancer cell lines. (A) IC_{50} values of TAB12 and TAB14 in MDA-MB-231, MCF7, CNE2, and Hep3B cells. (B, C) Dose-response curves for (B) TAB12 and (C) TAB14 in MDA-MB-231, MCF7, CNE2, and Hep3B cells. Cell viability was assessed using the CCK-8 assay after 24 h of peptide treatment.

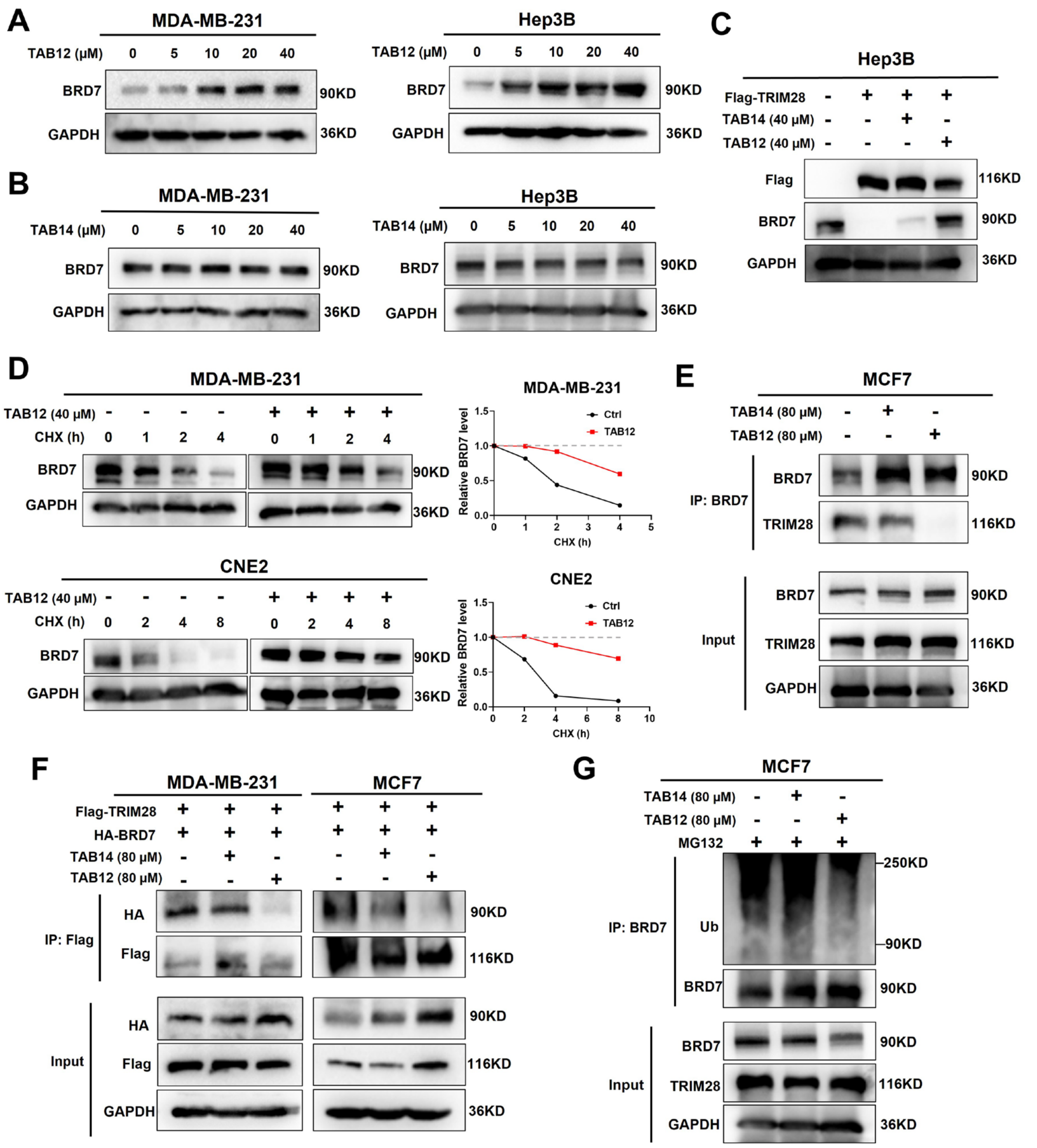

Figure S4 | Evaluation of the effects of TAB14 and TAB12 on BRD7 protein levels. (A, B) MDA-MB-231 and Hep3B cells were treated with (A) TAB12 or (B) TAB14 at concentrations of 0, 5, 10, 20, and 40 µM for 24 h. BRD7 expression levels were then detected by Western blot. (C) The effects of 40 µM TAB14 or TAB12 treatment for 24 h on BRD7 expression were examined in Hep3B cells overexpressing TRIM28. (D) After treatment with 40 µM TAB12 for 24 h, 50 µM CHX was added. BRD7 protein expression was detected by Western blot. BRD7 protein levels were quantitatively analyzed and plotted as a line graph. (E) MCF7 cells were treated with 80 µM TAB14 or TAB12 for 24 h. Co-IP assays were then performed using a BRD7 antibody to analyze the effects of peptides on the TRIM28-BRD7 protein interaction. (F) Following co-transfection with Flag-TRIM28 and HA-BRD7 plasmids and treatment with 80 µM peptides for 24 h, Co-IP experiments were carried out using an anti-Flag antibody to analyze the impact of TAB14 and TAB12 on the exogenous TRIM28-BRD7 interaction. (G) MCF7 cells were incubated with either 80 µM TAB14 or TAB12 for 24 h. The cell lysates were subjected to Co-IP using a BRD7 antibody, and the ubiquitination level of BRD7 was detected by Western blot with an Ub antibody.

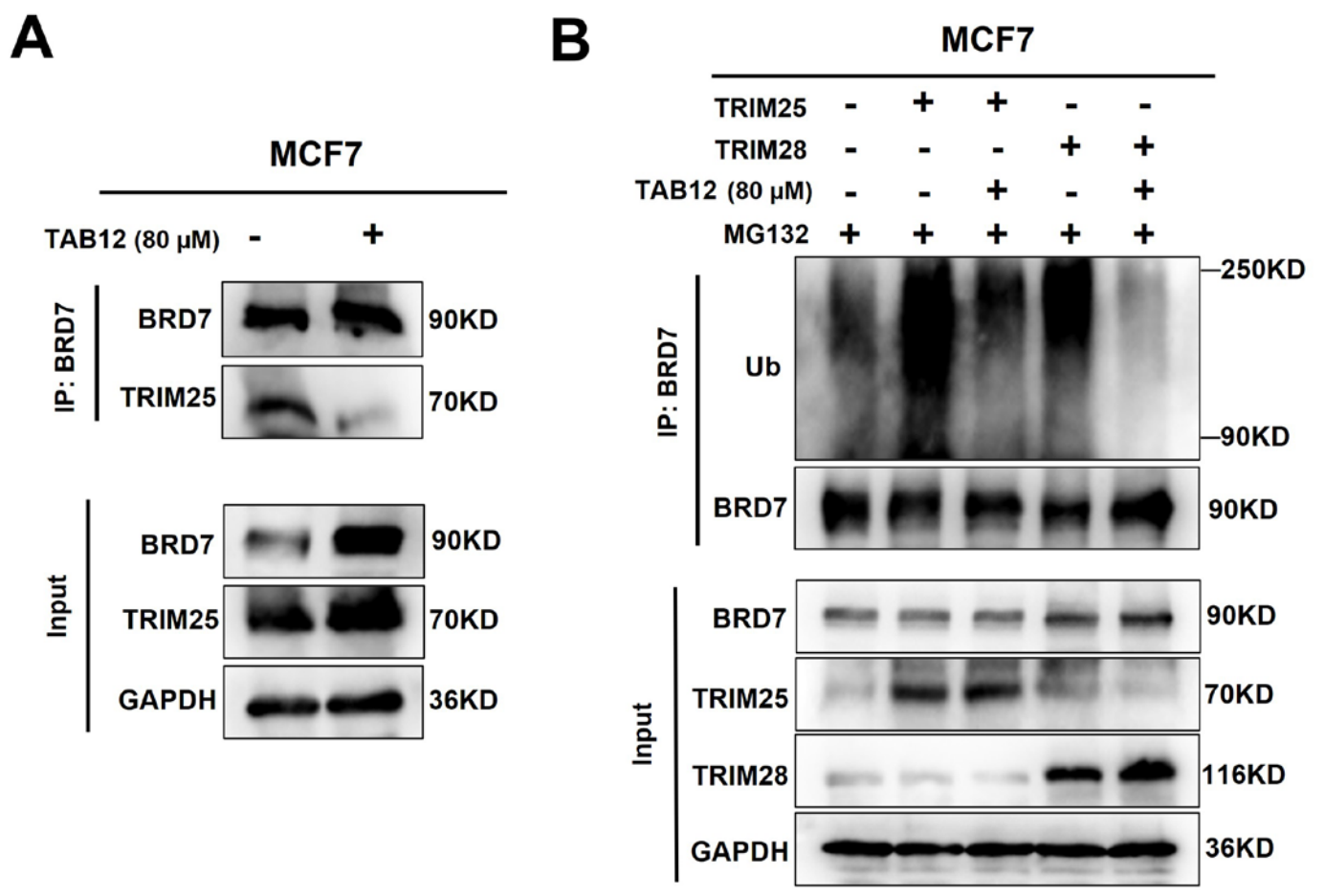

Figure S5 | Effects of TAB12 on the TRIM25-BRD7 interaction and TRIM28 binding to its interacting proteins. (A) MCF7 cells were treated with 80 μM TAB12 or vehicle control for 24 h and then harvested. Co-IP was performed using a BRD7 antibody to assess TRIM25 enrichment. (B) Following transfection with Vector, TRIM25, or TRIM28 expression plasmids, MCF7 cells were treated with 80 μM TAB12 or vehicle control for 24 h, with MG132 added for the final 4 h before harvest. Co-IP was performed with an anti-BRD7 antibody to evaluate BRD7 ubiquitination mediated by TRIM25 and TRIM28. (C) Co-IP analysis of interactions between TRIM28 and its interacting proteins (BRD7, Rb, p53, and p27) in cells treated with vehicle control or 80 μM TAB12.

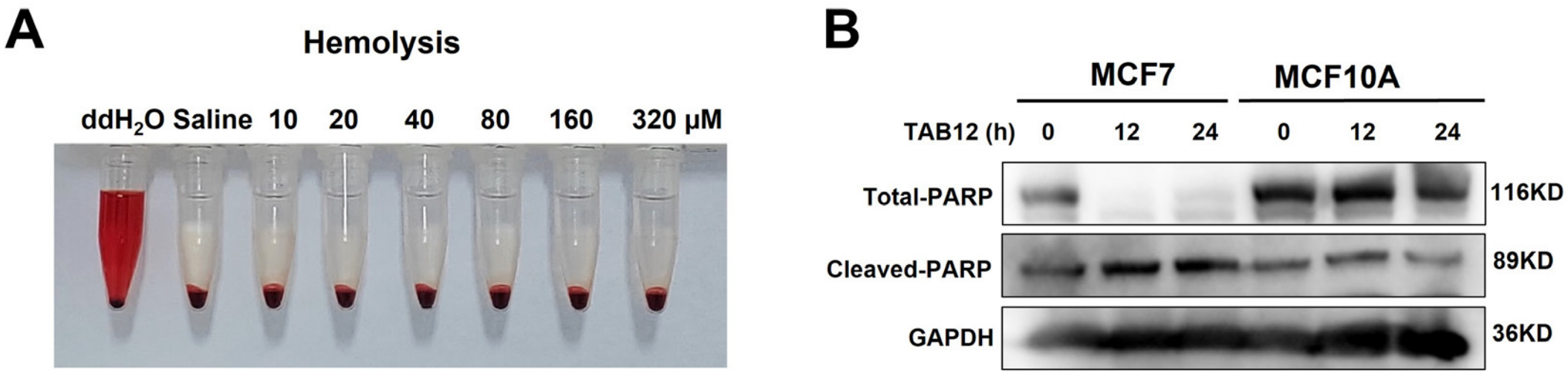

Figure S6 | Toxicity of peptide inhibitor TAB12 on normal cells and cancer cells. (A) Representative image of hemolysis assay of erythrocytes incubated with TAB12 at concentrations from 10 to 320 μM, with ddH_2O as the positive control and saline as the negative control. (B) Expression levels of Total-PARP and Cleaved-PARP were detected by Western blot in MCF7 and MCF10A cells after treatment with 80 μM TAB12 for 0, 12, or 24 h.

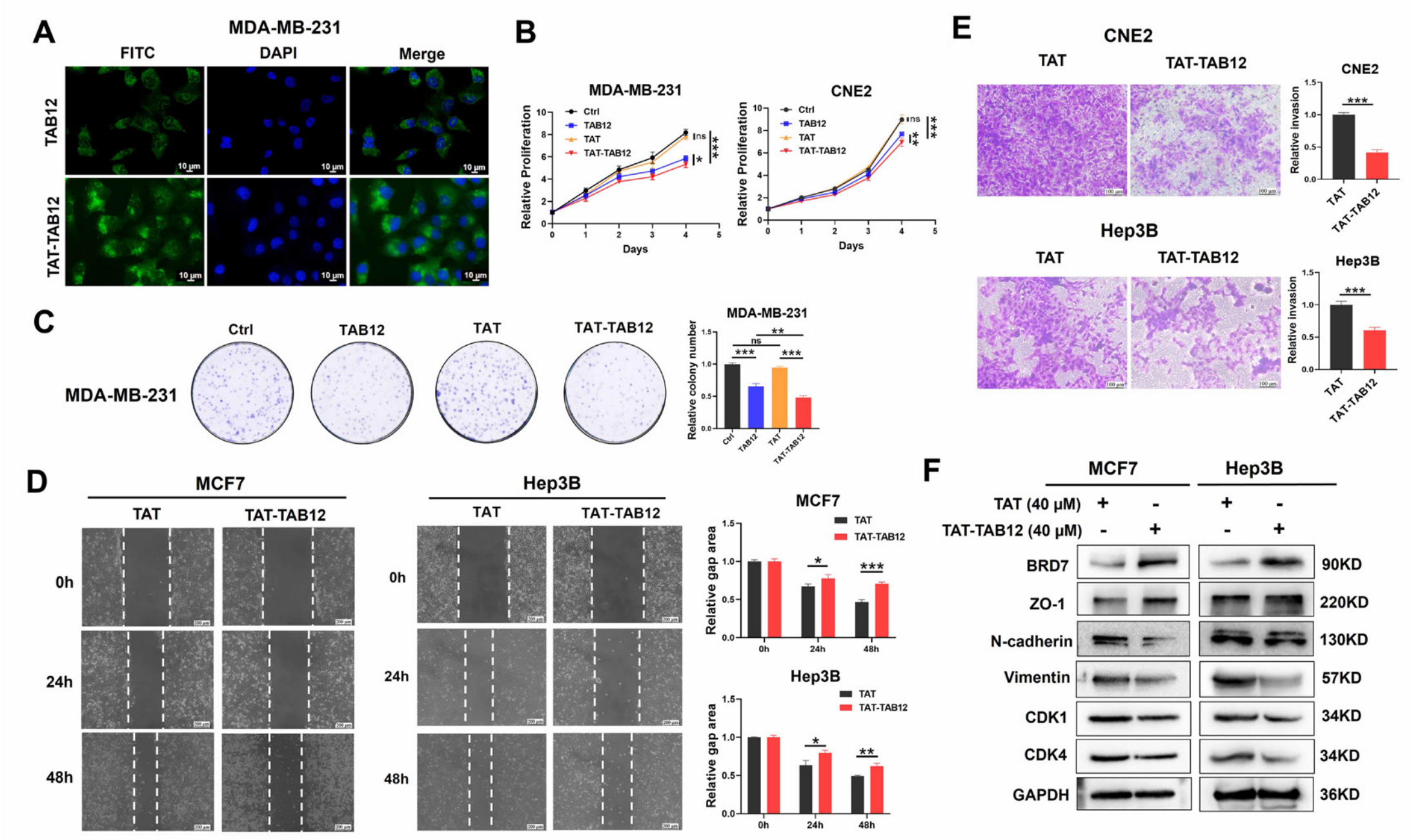

Figure S7 | TAT modification enhances the anti-tumor effects of TAB12. (A) MDA-MB-231 cells were incubated with 30 μM FITC-labeled TAB12 or TAT-TAB12 for 2 h, and cellular uptake was observed using fluorescence microscopy. The cell nuclei were stained with DAPI. Scale bar, 10 μm. (B) The impact of 80 μM TAB12 or TAT-TAB12 on the proliferation capacity of MDA-MB-231 and CNE2 cells was evaluated using the CCK-8 assay. (C) Colony formation assays were performed to evaluate the effects of 80 μM TAB12 or TAT-TAB12 on the colony-forming ability of MDA-MB-231 cells. (D) The migratory capacity of MCF7 and Hep3B cells following 80 μM TAT or TAT-TAB12 treatments was evaluated using the Wound healing assay. (E) The invasive potential of CNE2 and Hep3B cells after 80 μM TAT and TAT-TAB12 treatments was assessed using the Matrigel invasion assay. (F) The expression levels of BRD7, ZO-1, N-cadherin, Vimentin, CDK1, and CDK4 proteins in MCF7 and Hep3B cells treated with 40 μM TAT or TAT-TAB12 for 12 h were detected by Western blot. The data in (B, C, D, E) are presented as mean ± SD ($n \geq 3$). *, $P < 0.05$; **, $P < 0.01$; ***, $P < 0.001$; ns, no significance.

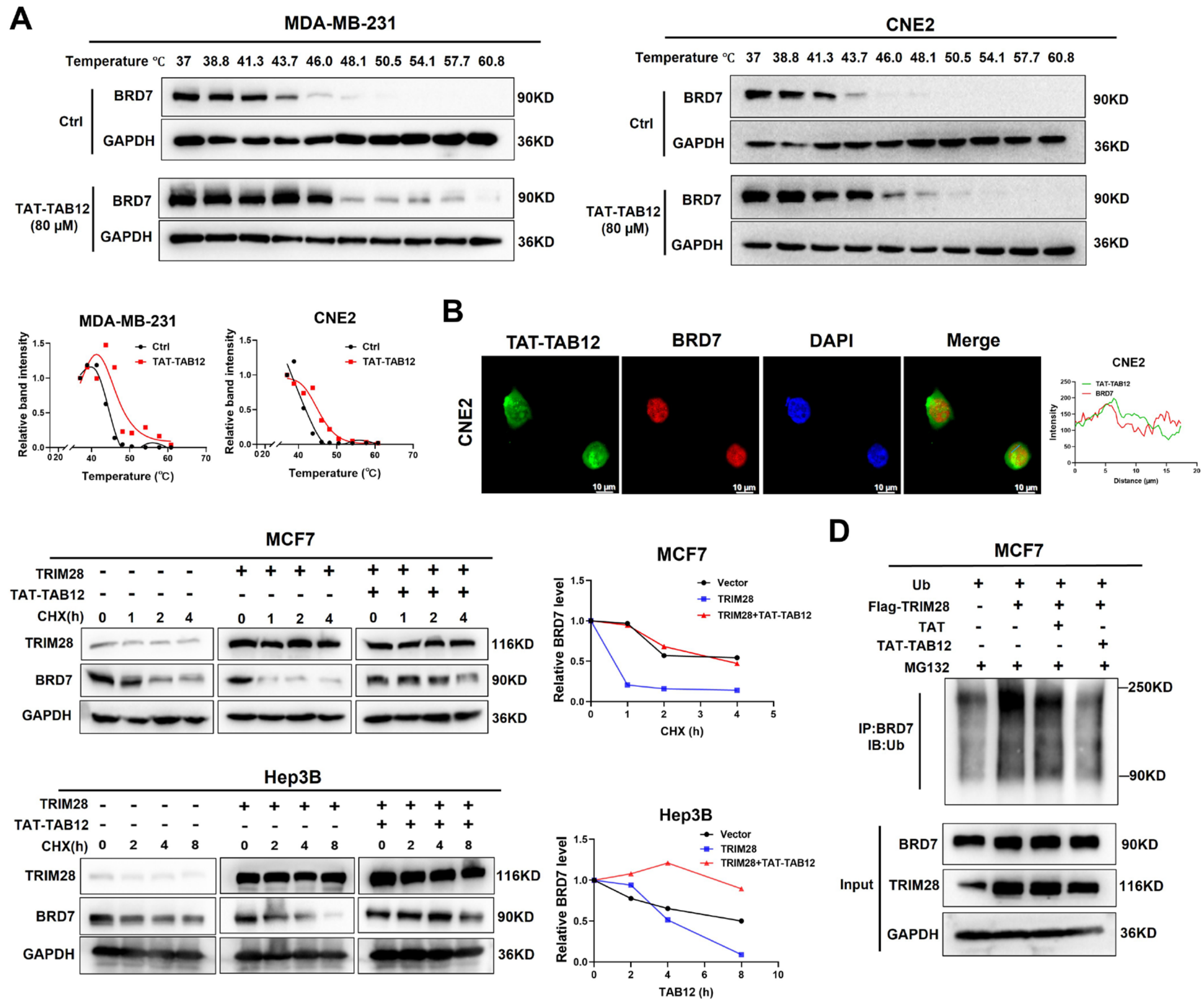

Figure S8 | Characterization of the interaction between TAT-TAB12 and BRD7 and its regulatory effects on BRD7 stability and ubiquitination. (A) MDA-MB-231 and CNE2 cells were incubated with 80 μM TAT-TAB12 or control for 12 h. CETSA was performed to evaluate the impact of the peptide on the thermal stability of BRD7. (B) After CNE2 cells were incubated with 30 μM TAT-TAB12 for 2 h, immunofluorescence analysis and confocal microscopy were used to examine the co-localization of TAT-TAB12 and BRD7. TAT-TAB12 was FITC-labeled, endogenous BRD7 was stained with an anti-BRD7 (red) antibody, and cell nuclei were stained with DAPI. Scale bar, 10 μm. (C) MCF7 and Hep3B cells overexpressing TRIM28 were treated with 40 μM TAT-TAB12 for 12 h, followed by the addition of 50 μM CHX at various time points. The expression level of BRD7 was detected by Western blot, and the protein expression of BRD7 was further quantitatively analyzed and represented with a line graph. (D) After co-transfection with Vector or TRIM28 and Ub, cells were treated with 80 μM TAT or TAT-TAB12 for 12 h. A ubiquitination assay was then performed to detect BRD7 ubiquitination levels.

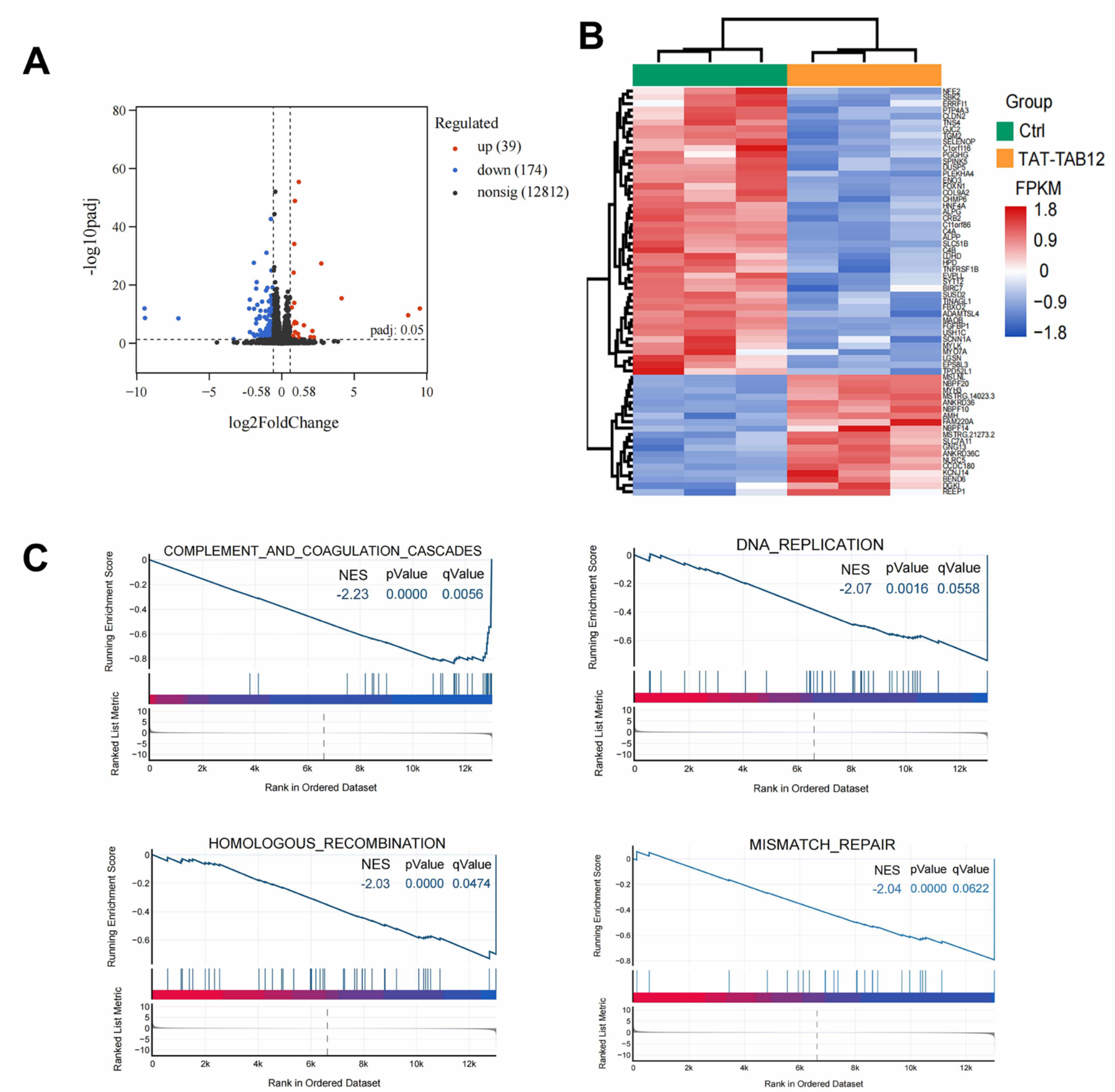

Figure S9 | RNA-Seq analysis of MCF7 cells treated with TAT-TAB12. (A) Volcano plot of differentially expressed genes (DEGs) in control and 80 μM TAT-TAB12-treated cells. Genes with $|\log_2 FC| \geq 0.58$ (1.5-fold) and adjusted P value < 0.05 were defined as differentially expressed. (B) Clustering heatmap of DEGs. (C) Gene set enrichment analysis (GSEA) was performed using the ranked gene list from the comparison between control and TAT-TAB12-treated cells, with $|NES| > 1$ and FDR < 0.25 set as the significance threshold.

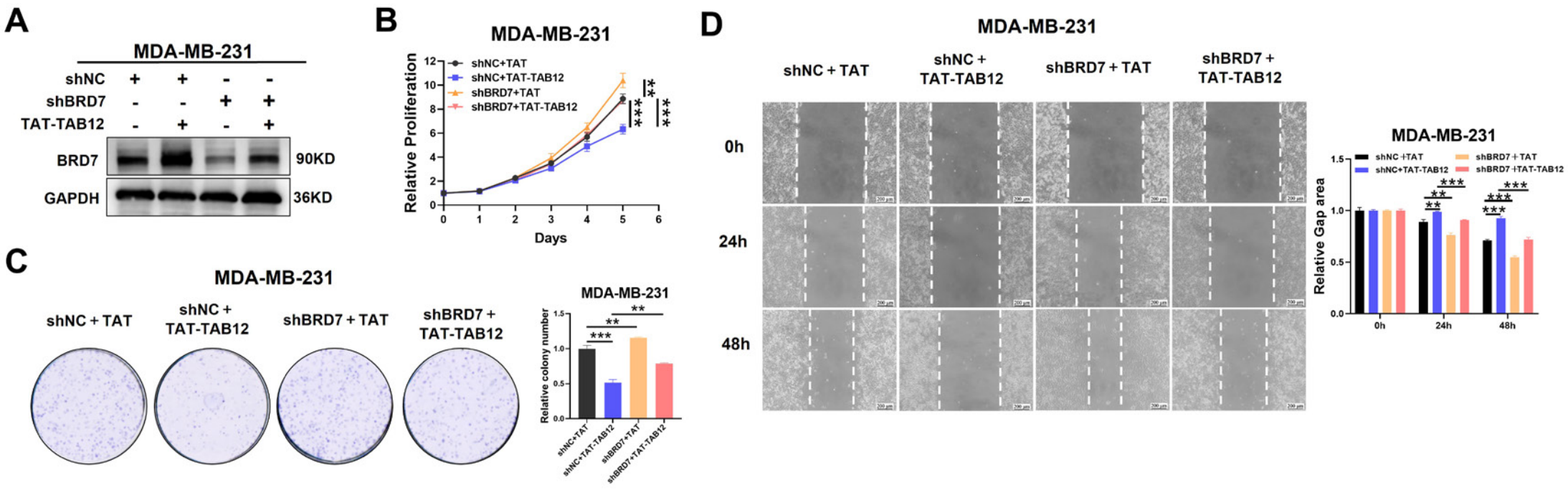

Figure S10 | TAT-TAB12 inhibits tumor progression by stabilizing the BRD7 protein. (A) BRD7 was stably knocked down in MDA-MB-231 cells using shBRD7, with shNC as a control. These cells were then treated with 80 μM TAT or TAT-TAB12 for 12 h. Western blot assay was performed to detect BRD7 expression levels. (B–D) Stable BRD7 knockdown (shBRD7) and control (shNC) cells were treated with 80 μM TAT or TAT-TAB12. The effects of TAT and TAT-TAB12 on cell proliferation, colony formation, and migration with or without BRD7 knockdown were explored via (B) CCK-8 assay, (C) colony formation assay, and (D) wound healing assay. The data in (B, C, D) are presented as mean ± SD ($n \geq 3$). *, $P < 0.05$; **, $P < 0.01$; ***, $P < 0.001$; ns, no significance.

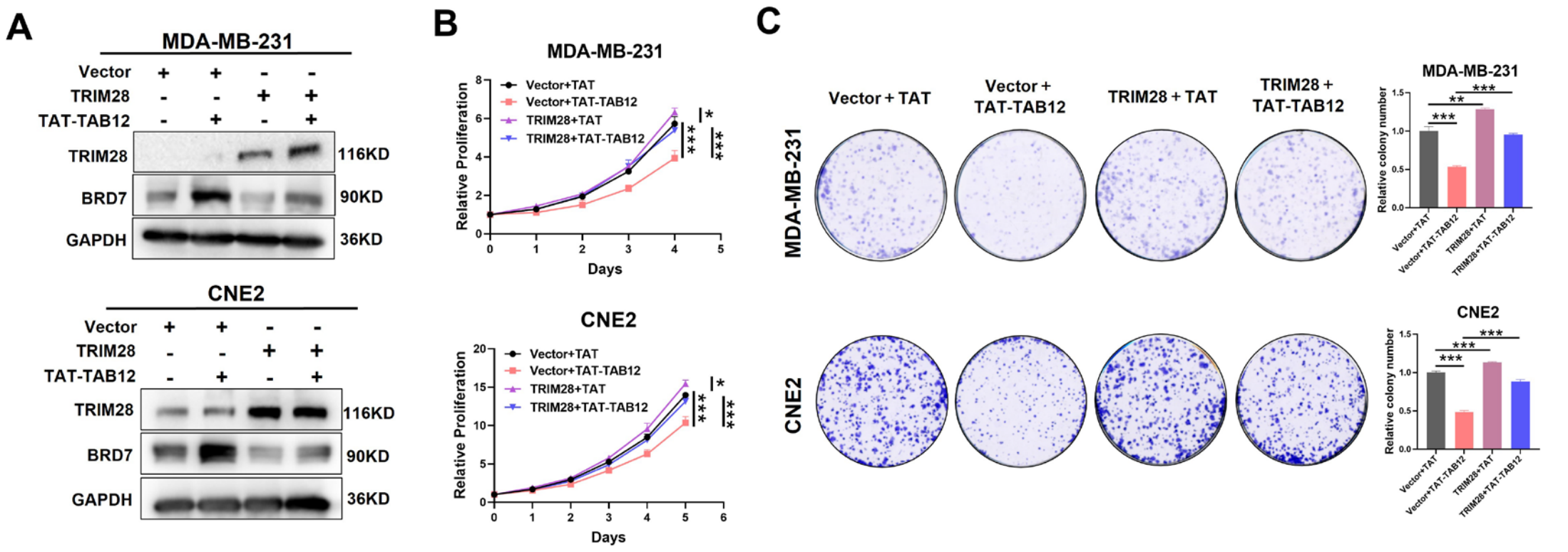

Figure S11 | TAT-TAB12 exerts its anti-tumor activity by competing with TRIM28 for BRD7. (A) MDA-MB-231 and CNE2 cells were transfected with Vector or TRIM28 and then treated with 80 μM TAT or TAT-TAB12 for 12 h. Western blot assay was performed to detect TRIM28 and BRD7 expression levels. (B, C) The effects of 80 μM TAT and TAT-TAB12 on cell proliferation and colony formation with or without TRIM28 overexpression were evaluated by (B) CCK-8 assay and (C) colony formation assay. The data in (B, C) are presented as mean ± SD (n ≥ 3). *, P < 0.05; **, P < 0.01; ***, P < 0.001.

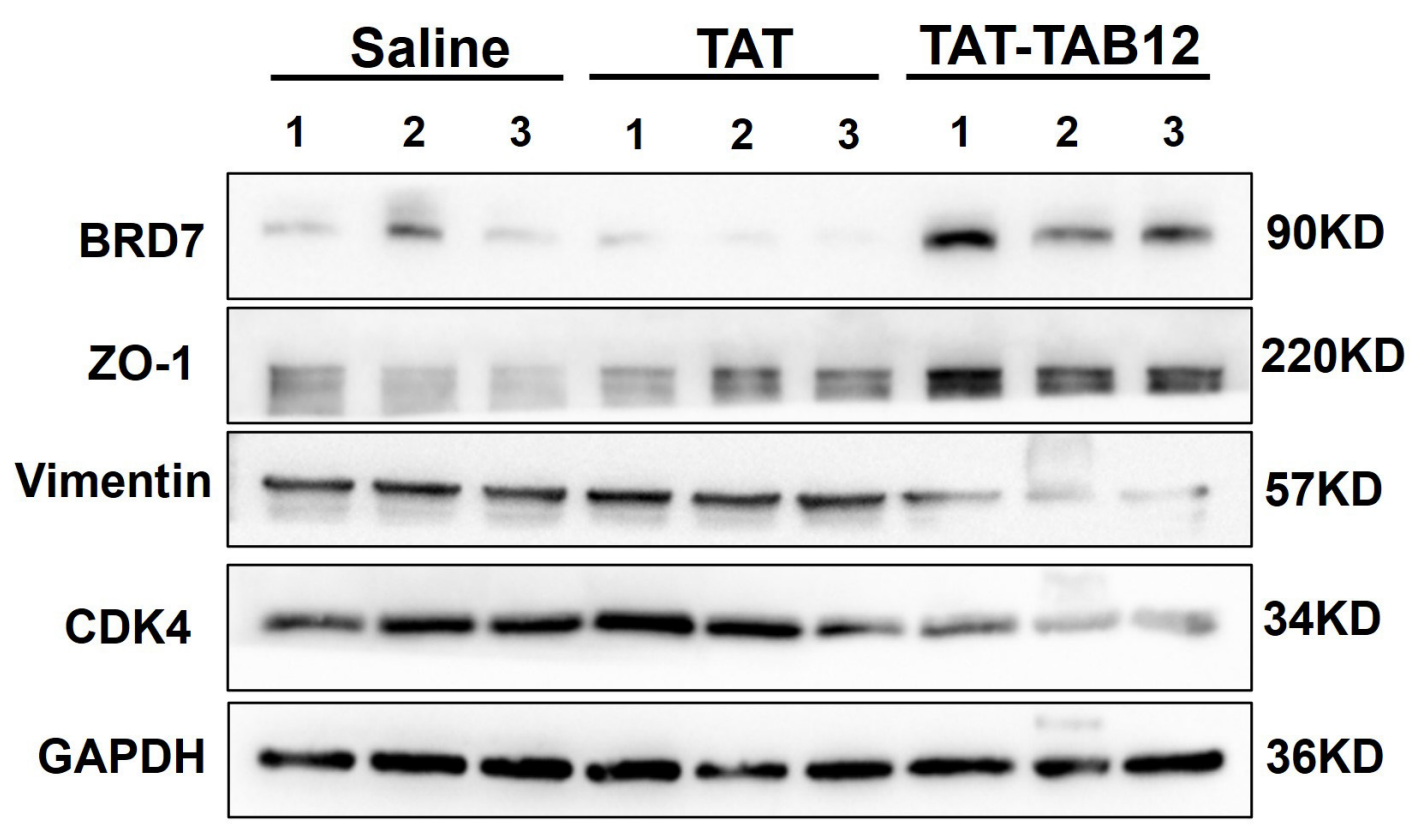

Figure S12 | The expression levels of relevant molecules in xenograft tumor tissues. Three tumor tissues were randomly selected from each group (saline, TAT, and TAT-TAB12), and the expression levels of BRD7, ZO-1, Vimentin, and CDK4 were detected by Western blotting.
